# Caplacizumab use in immune-mediated thrombotic thrombocytopenic purpura: an international multicentre retrospective Cohort study (The Capla 1000+ project)

**DOI:** 10.1016/j.eclinm.2025.103168

**Published:** 2025-03-30

**Authors:** Paul Coppo, Michael Bubenheim, Ygal Benhamou, Linus Völker, Paul Brinkkötter, Lucas Kühne, Paul Knöbl, Maria Eva Mingot-Castellano, Cristina Pascual-Izquierdo, Javier de la Rubia, Julio del Rio Garma, Shruti Chaturvedi, Camila Masias, Marshall Mazepa, X. Long Zheng, György Sinkovits, Marienn Réti, Christopher J. Patriquin, Katerina Pavenski, Tiago Boechat, João Farias, Eduardo Flavio Oliveira Ribeiro, Michaela Larissa Lobo de Andrade, Agnès Veyradier, Bérangère Joly, Raïda Bouzid, Kazuya Sakai, Masanori Matsumoto, Pasquale Agosti, Ilaria Mancini, Flora Peyvandi, Eleni Gavriilaki, Matthew Stubbs, Amjad Hmaid, Spero Cataland, Bernhard Lämmle, Marie Scully

**Affiliations:** a Centre de Référence des Microangiopathies Thrombotiques, Service d’Hématologie, Assistance Publique–Hôpitaux de Paris (AP-HP) and Sorbonne Université, Paris, France; b INSERM Unité Mixte de Recherche (UMRS) 1138, Centre de Recherche des Cordeliers, Paris, France; c Department of Clinical Research and Innovation, Centre Hospitalier Universitaire (CHU) de Rouen; d Département de Médecine Interne, CHU Charles Nicolle, Rouen, France; e Normandie University, UNIROUEN, INSERM U1096 EnVI, Rouen, France; f Department II of Internal Medicine and Center for Molecular Medicine Cologne, Faculty of Medicine and University Hospital Cologne, University of Cologne, Germany; g Cologne Cluster of Excellence on Cellular Stress Responses in Ageing-Associated Diseases, Cologne, Germany; h Division of Hematology and Hemostasis, Department of Medicine 1, Medical University of Vienna, Austria; i Servicio de Hematología, Hospital Universitario Virgen del Rocío, Instituto de Biomedicina de Sevilla, Universidad de Sevilla, Spain; j Servicio de Hematología, Hospital Universitario Gregorio Marañón, Instituto de Investigación Gregorio Marañón, Madrid, Spain; k Servicio de Hematología, Hospital Universitario y Politécnico La Fe, Valencia & Universidad Católica de Valencia, Spain; l Complexo Hospitalario Universitario de Santiago, Spain; m Department of Medicine, Johns Hopkins University, Baltimore, MD, USA; n Baptist Health South Florida, Miami, FL, USA; o Department of Medicine, University of Minnesota, Minneapolis, MN, USA; p Department of Pathology and Laboratory Medicine, University of Kansas Medical Center, Kansas City, KS, USA; q Department of Internal Medicine and Haematology, Semmelweis University, Budapest, Hungary; r Department of Hematology and Stem Cell Transplantation, Central Hospital of Southern Pest - Institute of Hematology and Infectious Diseases, Budapest, Hungary; s Department of Hematology, University Health Network, Toronto, Canada; t Department of Laboratory Medicine, St. Michael's Hospital, Toronto, Canada; u Department of Hematology - Hematology and Hemotherapy Institute of Rio de Janeiro (HEMORIO), Rio de Janeiro, RJ, Brazil; v Department of Hematology, Hospital Erasto Gaertner, Curitiba, Brazil; w Santa Lucia Hospital, Brasilia, DF, Brazil; x Hématologie Biologique, Hôpital Lariboisière, AP-HP, Paris, France; y Department of Blood Transfusion Medicine, Nara Medical University, Kashihara, Japan; z Università degli Studi di Milano, Department of Pathophysiology and Transplantation and Fondazione Luigi Villa, Milan, Italy; aa Fondazione IRCCS Ca’ Granda Ospedale Maggiore Policlinico, Angelo Bianchi Bonomi Hemophilia and Thrombosis Center, Milan, Italy; ab BMT Unit – Department of Hematology, G. Papanicolaou Hospital, Thessaloniki, Greece; ac University College London Hospitals NHS Foundation Trust, London, UK; ad Department of Internal Medicine, Ohio State University, Columbus, OH, USA; ae Center for Thrombosis and Hemostasis, University Medical Center of Johannes Gutenberg University, Mainz, Germany; af Department of Hematology and Central Hematology Laboratory, Inselspital, Bern University Hospital, University of Bern, Bern, Switzerland

**Keywords:** Thrombotic thrombocytopenic purpura, Caplacizumab, ADAMTS13, Rituximab, Prognosis

## Abstract

**Background:**

The anti-Von Willebrand Factor (VWF) nanobody caplacizumab is licensed for adults with immune-mediated thrombotic thrombocytopenic purpura (iTTP) in association with therapeutic plasma exchange (TPE) and immunosuppression. However, whether caplacizumab reduces mortality, and its optimal timing of initiation, is not completely settled.

**Methods:**

This international, multicenter retrospective cohort study recruited patients from 2018 until 2023 and data collection took place from January 1st to June 30th 2023 in the participating centers. One thousand and fifteen patients were treated with daily TPE, immunosuppression with corticosteroids ± rituximab, and caplacizumab (caplacizumab group), which was compared to historic controls treated with TPE and corticosteroids ± rituximab (control group, N = 510). Caplacizumab initiation was classified as early (within 3 days; 76% of cases) or delayed (≥4 days from first TPE).

**Findings:**

Three-month survival rate in the caplacizumab group was 98.5%, compared with 94% in controls (P < 0.0001). Three-month mortality rate was 4.2-fold higher in controls than in caplacizumab-treated patients (95% CI: 2.22–7.7, P < 0.0001), regardless of rituximab use. In both groups, death was observed primarily in elderly patients, and age was the factor most associated with 3-month mortality. Patients receiving caplacizumab showed reduced refractoriness, exacerbations, and required fewer TPE sessions to achieve clinical response versus controls (P < 0.0001 all). Time to clinical response in the caplacizumab group was shorter than in controls, and even shorter in patients with early caplacizumab initiation (P < 0.0001 both). Caplacizumab-related adverse events were observed in 21% of patients, with major bleeding in 2.4%, which was more common in elderly patients.

**Interpretation:**

The early association of Caplacizumab to TPE and immunosuppression significantly reduces unfavorable outcomes during iTTP, including death, and alleviates the burden of care at the potential expense of bleeding events. Advanced age, however, remains an adverse factor for survival. The limitations of our study include its retrospective and multicentric design and the use of a historical control cohort.

**Funding:**

None.

**Research in context**

**Evidence before this study**

iTTP remained an invariably fatal disease until the systematic use of therapeutic plasma exchange (TPE) with corticosteroids that allowed reaching survival rates of 80–85%. Later, the addition of the B-cell depleting agent rituximab accelerated clinical responses and reduced relapses; however, as rituximab efficacy is observed only after at least two weeks following the first administration, it could not reduce early mortality or exacerbations that typically occur within the first few days or weeks of treatment, respectively. The anti-VWF nanobody caplacizumab directed against the A1 domain of VWF was licensed for adults with iTTP as part of the standard treatment. We searched PubMed with the keywords “caplacizumab” “thrombotic thrombocytopenic purpura”, for articles published in English from 2016, the date of the first clinical trial reporting the efficacy of caplacizumab, to 2024. We identified 284 articles and decided to focus mostly on papers involving randomized controlled trials and subsequent post-hoc analyses, phase 1/2 studies, real-world experience studies that recruited at least 10 patients and meta-analyses, which represents roughly 35 articles. These works have provided evidence that caplacizumab added to TPE and immunosuppression improved the outcome of the acute phase of the disease at the expense of adverse events, mostly consisting of manageable muco-cutaneous bleeding. However, pivotal trials were not designed to demonstrate a survival benefit and so far, only integrated analyses of pivotal trials as well as meta-analyses including real-life studies suggested that caplacizumab was associated with decreased mortality. Hence, whether caplacizumab can truly reduce mortality is still not completely settled. In addition, the optimal timing of caplacizumab initiation, i.e., its use as a frontline in all patients or as a salvage therapy, also remains debated.

**Added value of this study**

We report here the largest international series of caplacizumab-treated patients (N = 1015), with four main findings. We show additional evidence that caplacizumab, added to daily TPE and immunosuppression, improves prognosis of acute iTTP by preventing refractoriness and exacerbations, with a 4-fold reduction of 3-month death risk. We found a particularly low number of patients needed to treat with caplacizumab to prevent any unfavorable outcome (i.e., less than 4 patients). From a large number of patients, our results further support that adverse events are usually manageable. Apart from age, we could not identify clear predictors of mortality, especially in the caplacizumab group. Hence, a delayed introduction of caplacizumab during the management may expose patients to a higher risk of unpredictable refractoriness and exacerbation, which may precede death. We also found that the early use of caplacizumab substantially alleviated the burden of care, with a decrease in time to clinical response and in the number of needed TPE sessions when compared to controls. We observed that non-survivors in the caplacizumab group were more frequently elderly patients who succumbed later from underlying comorbidities and from complications related to hospitalization while iTTP was in clinical response, suggesting that caplacizumab has changed the pattern of death in iTTP that now predominantly occurs in patients with frailty and comorbidities. Our study has a solid methodology: the participation of expert clinicians and biologists from 12 university hospitals worldwide; two cohorts of caplacizumab-treated and control patients recruited on the basis of stringent inclusion criteria according to the updated definition of thrombotic thrombocytopenic purpura—i.e., thrombotic microangiopathy with no associated condition and with severe ADAMTS13 (A Disintegrin And Metalloproteinase with ThromboSpondin type 1 motifs; 13th member) deficiency; high-quality monitoring of clinical data by expert teams; and a management of patients by experienced teams according to the most recent standards.

**Implications of all the available evidence**

This international academic effort on such a rare disease provides additional evidence that caplacizumab improves iTTP prognosis in the acute phase by achieving impressive survival rates of up to 98.5% while decreasing the TPE-related burden of care. Our findings are important because they strongly argue for a systematic use of caplacizumab early in the management, typically as soon as the diagnosis of iTTP and the decision to start TPE are made, to prevent the formation of deleterious microthrombi and prevent adverse outcomes. Efforts are now required to evaluate TPE-free or -alleviated regimens, but also more efficient immunomodulation regimens in slow responders to standard immunosuppression to offset the cost burden of prolonged use of caplacizumab. New strategies to better control age-related comorbidities and prevent post-iTTP complications are also needed with caplacizumab-containing regimens to further improve the prognosis of the disease.

## Introduction

Immune-mediated thrombotic thrombocytopenic purpura (iTTP) is caused by autoantibodies directed against the plasma Von Willebrand factor (VWF)-cleaving protease ADAMTS13 (A Disintegrin And Metalloproteinase with ThromboSpondin type 1 motifs; 13th member), resulting in severe deficiency of ADAMTS13 activity. In this context, ultra-large VWF multimers accumulate on endothelial surfaces and in the circulation, leading to an increased platelet clumping in arterioles and microvasculature. Patients with iTTP present with a severe consumptive thrombocytopenia, microangiopathic hemolytic anemia, and life-threatening ischemic organ damage.^1^

iTTP remained an invariably fatal disease until the systematic use of therapeutic plasma exchange (TPE) with corticosteroids that allowed reaching survival rates of 80–85%.^2^, ^3^, ^4^ Later, the addition of the B-cell depleting agent rituximab accelerated clinical responses and reduced relapses; however, as rituximab efficacy is observed only after at least two weeks following the first administration,^5^^,^^6^ it would not be expected to reduce early mortality or exacerbations that typically occur within the first few days or weeks of treatment, respectively.^6^^,^^7^

The anti-VWF nanobody caplacizumab (Cablivi™) directed against the A1 domain of VWF is licensed for adults with iTTP as part of the standard treatment. Prospective controlled trials and national real-world studies have provided evidence that caplacizumab added to TPE and immunosuppression improved the outcome of the acute phase of the disease at the expense of adverse events, mostly consisting of manageable muco-cutaneous bleeding.^8^^,^^9^ However, pivotal trials were not designed to demonstrate a survival benefit. Moreover, patients at high risk of bleeding were excluded from these studies, precluding a comprehensive description of adverse events.^8^^,^^9^ So far, only integrated analyses^10^^,^^11^ of pivotal trials as well as meta-analyses including real-life studies suggested that caplacizumab was associated with decreased mortality. Hence, whether caplacizumab can truly reduce mortality is still not completely settled. In addition, the optimal timing of caplacizumab initiation, i.e., its use as a frontline in all patients or as a salvage therapy, also remains debated.^12^^,^^13^ Lastly, rare cases of serious bleeding complications including intracerebral hemorrhage were reported,^14^ which hints at more cautious use in patients with a higher risk of bleeding.^15^ To address these important issues, an international survey, the Capla 1000+ project, has been conducted.

## Methods

### Study design and data collection

As an initiative from the International Working Group on TTP (IWG-TTP),^16^^,^^17^ a worldwide academic call was launched to teams with experience in the use of caplacizumab to gather information on patients treated with a therapeutic regimen including caplacizumab, TPE and immunosuppression with corticosteroids ± rituximab (caplacizumab group). A minimal set of harmonized items was defined by the four investigators who initiated this work (PC, SC, MS and BL) and submitted to all invited participants to collect anonymized standard care data (Supplemental File 1).

The primary outcome was 3-month survival post-first TPE, considered as the period of time allowing to capture exhaustively iTTP-related deaths. Key secondary outcomes were refractoriness and exacerbations, time to clinical response from first TPE (defined as a sustained platelet count ≥150 × 10^3^/mm^3^; Supplemental Table S1), number of TPE to achieve clinical response, time from first TPE to sustained ADAMTS13 activity recovery ≥20% on weekly assessments post-TPE (i.e., ADAMTS13 response),^17^ and caplacizumab-related adverse events.

To assess the impact of the schedule of caplacizumab initiation on the above-mentioned outcomes, we compared patients with early initiation, i.e., when started within three days from first TPE, to those with delayed initiation, i.e., when started from day 4.

In order to address improvements in the disease burden due to caplacizumab, a cohort of iTTP patients not treated with caplacizumab (control group) was formed by gathering the same clinical data as for the caplacizumab group. For consistency in the management, information was collected on a 1-to-2 ratio for patients managed during the most recent years before the systematic use of caplacizumab, i.e., from 2015 to 2018, mainly from the teams that recruited most of the patients treated with caplacizumab (i.e., France, United Kingdom, Germany, Spain and United States) (see Supplemental Material).

This work was designed to comply with the reporting guidelines as given by the STROBE (Strengthening the reporting of observational studies in epidemiology) statement (Strengthening the reporting of observational studies in epidemiology [strobe-statement.org]) (Supplemental File 2).

### Patient recruitment and outcomes

Each participating team retrieved records of patients with a confirmed diagnosis of iTTP, who received at least one TPE in association with caplacizumab, with available data to a minimum of 3-month follow-up post-first TPE unless death occurred, as per the primary endpoint. The period of recruitment started from the date caplacizumab was licensed in most countries (i.e., 2018 in the European Union and 2019 in the United States) until 2023. Data collection took place from January 1st to June 30th 2023 in the participating centers. Diagnostic criteria and assessment of severity and outcomes are detailed in Supplemental Methods.

### Treatment

Treatment with caplacizumab was started either when the diagnosis of iTTP was suspected based on clinical judgement and predictive scores (the French score or the PLASMIC score depending on the use of each team), or when it was confirmed within 24 h by a severely decreased ADAMTS13 activity. As per inclusion criteria, all patients received daily TPE, and usually corticosteroids with rituximab (detailed in Supplemental Methods). Caplacizumab (10 mg intravenous loading dose followed by daily 10 mg subcutaneous doses) was added to TPE and immunosuppression on a variable schedule, i.e., rapidly after TPE initiation (defined here as within three days following first TPE), or delayed (≥4 days following first TPE). The period of continuation of caplacizumab was as per investigator's choice, but followed the pathway presented in the pivotal study^8^ i.e., given for a 30-day period that could be extended if ADAMTS13 activity remained depressed for up to four additional weeks or longer, pending ADAMTS13 improvement (defined by an activity ≥20%).^17^, ^18^, ^19^

### Ethics

All patients provided their consent for the reuse of standard of care data, according to the regulatory rules of each country. This study was conducted in accordance with the Declaration of Helsinki, and the Data Protection Authority of each country. Where required, consent was sought and local research ethic board approval was obtained, and a data transfer agreement (available on request) was signed between the team issuing the data and the one receiving them, i.e., the French team.

### Statistics

Cohorts are described by absolute numbers for nominal variables and otherwise by the median with 1st and 3rd quartiles [Q1–Q3]. To assess whether cohorts differed at TPE start, the rank-based Mann-Whitney-Wilcoxon test was used for variables of at least ordinal level and Freeman-Halton's test otherwise. All presented odds ratios and their respective 95% confidence intervals (95% CI) were obtained via logistic regression. To assess whether caplacizumab was independently associated with 3-month survival, we performed a logistical regression. For this, we compared by univariable analysis 3-month survivors and non-survivors for historical predictive factors of death and treatment modalities. Variables significantly associated with mortality were included in a backward multivariable analysis. Only variables at P = 0.05 were kept in the final model.

The figure on mortality was obtained using Kaplan–Meier's method and differences between cohorts were assessed with the Log–Rank test. Figures on the occurrence of clinical response, and exacerbation up to 30 days after the last TPE, show the cumulative probability of obtaining these endpoints in the presence of the competing risk, i.e., death, and both cohorts were compared using the hazard ratio (HR) postulating a Cox model. We tested that variables realizations stemmed from a normal distribution by using the Kolmogorov–Smirnov test. Analyses were performed using the SAS 9.4 statistical software (SAS institute Inc, Cary, NC). The number of patients needed to treat to prevent one unfavorable event was calculated as the inverse of the absolute risk reduction, i.e., 1/(control group event rate–caplacizumab group event rate).

### Role of the funding source

This work received no financial support.

## Results

### Population characteristics and therapeutic groups

**Fig. 1:**
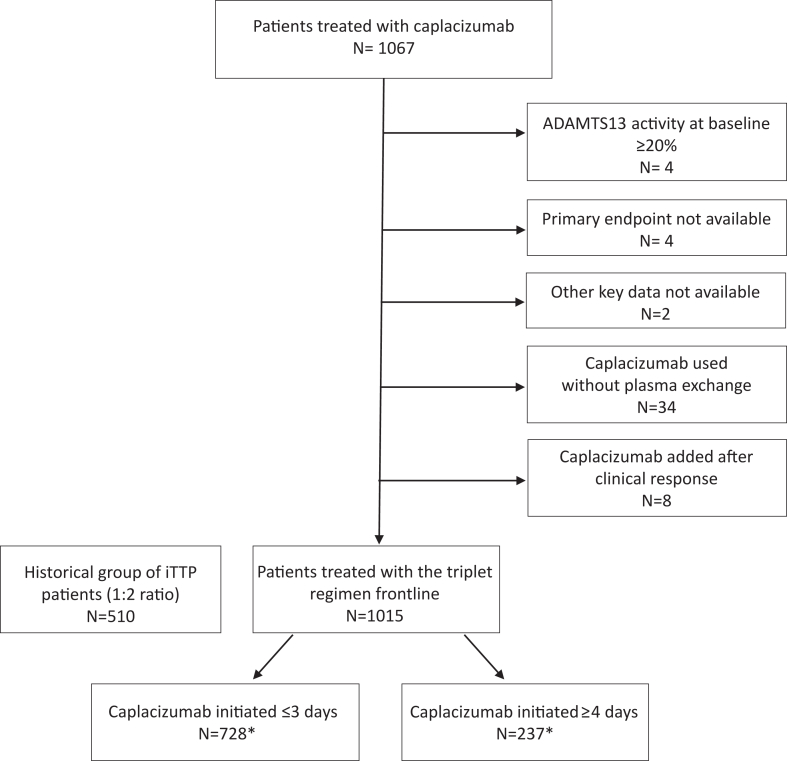
Flow chart of the study. iTTP, immune-mediated Thrombotic Thrombocytopenic Purpura. Date of caplacizumab initiation was missing for 50 patients.

Between January and June 2023, 1067 consecutive charts of iTTP patients treated with a caplacizumab-containing regimen were collected from 12 countries. Fifty-two patients were subsequently excluded (Fig. 1), leaving 1015 eligible patients. Among these, 396 (38%) were previously published (Supplemental Table S2).

These patients were treated primarily between 2018 and 2023 (98.7%); they received TPE, in association in most cases with corticosteroids and rituximab frontline or as salvage therapy as per practitioner's choice. Caplacizumab was started for 728 patients (72%) within 3 days and at least half of them received it on the day of their first TPE (Q1–Q3, 0–1). For 237 patients (23%), caplacizumab was started ≥4 days (median, 8 days [Q1–Q3, 4–11]) from first TPE (the date of caplacizumab initiation was missing for 50 patients). In the 237 patients with a later initiation (≥4 days), caplacizumab was added for the management of an exacerbation or for refractory iTTP (N = 90/237 cases, 38%). In 147 additional patients, caplacizumab initiation was delayed because of non-immediate availability of the agent or as per practitioner's choice.

Patients treated with caplacizumab were compared to 510 historical controls (Supplemental Table S2) (see Flow-chart; Fig. 1); rituximab was administered in 362 patients experiencing refractoriness or exacerbation (N = 278 cases), or frontline (United Kingdom group; N = 84 cases).

### Clinical presentation

Seventy-six to 80% of patients of both the caplacizumab and control groups had a French score of 2, consistent with the clinical suspicion of iTTP. Clinical presentation and severity were generally comparable between groups. Ethnicity differed between groups, with a higher prevalence of patients of African ancestry or originating from West Indies in the caplacizumab group (P < 0.0001) (Table 1). Confusion and focal neurologic deficiency were more prevalent in patients in the caplacizumab group (P = 0.03 and P < 0.0001, respectively); consequently, patients with a French severity score of ≥3 and considered at higher risk of death^7^ were more prevalent in the caplacizumab group (Table 1).

**Table 1:** Clinical presentation at diagnosis according to therapeutic groups.

	Caplacizumab group (N = 1015)	Control group (N = 510)	P-value
**Clinical presentation**			
Age (yo)	46 (33–58)	43 (33–56)	0.32
Elderly patients (>60 yo)	20%	17%	0.14
Females	67%	68.6%	0.59
Number of previous iTTP episodes			
None	81%	81%	0.87
1	14%	18%	
≥2	5%	1%	
Ethnicity			<0.0001
Caucasian	61.7%	77.4%	<0.0001
African—West Indies ancestry	31.6%	19%	<0.0001
Others	6.7%	3.6%	–
Neurologic involvement	65%	58%	0.01
Headache	37.3%	31%	0.03
Confusion	26.6%	18.7%	0.03
Seizure	8.5%	9%	0.74
Coma (Glasgow Coma Scale ≤8)	4.9%	4%	0.53
Focal deficiency	38.7%	22.2%	<0.0001
Troponin > upper normal value	71.6%	68.3%	0.50
Hemoglobin level (g/dL)	8.4 (7–10)	8.6 (7.3–10.4)	0.09
Platelet count (×10^9^/L)	12 (8–21)	13 (8–25)	0.06
Serum creatinine (μmol/L)	93 (72–124)	88 (72–121)	0.19
Estimated glomerular filtration rate (ml/min/1.73 m^2^)	74 (52–97)	75 (55–98)	0.21
LDH level (× upper normal value)	4 (2.3–6)	3.7 (2.3–6)	0.98
French diagnostic score^a^			
2	80%	75.7%	0.13
1	19%	22.3%	
0	1%	2%	
French severity score^b^	(N = 1002)	(N = 503)	0.012
Low (0–1)	572 (57%)	328 (65%)	
Intermediate (2)	280 (28%)	123 (24%)	
High (3–4)	150 (15%)	52 (11%)	
ADAMTS13 activity	<5% (<5%–<5%)	<5% (<5%–<5%)	–
Detectable free anti-ADAMTS13 antibodies	91%	85%	0.001
**Treatment**			
Therapeutic plasma exchange	100%	100%	–
Corticosteroids	99.2%	94%	<0.0001
Rituximab	90.5%	71%	<0.0001

Abbreviations: iTTP, immune-mediated thrombotic thrombocytopenic purpura; LDH, lactate dehydrogenase; ADAMTS13, A Disintegrin And Metalloproteinase with ThromboSpondin-1 motifs; member 13.

a French diagnostic score of 2: platelet count <30 × 10^3^/mm^3^ and serum creatinine <200 μmol/L (2.27 mg/dL); score of 1: platelet count <30 × 10^3^/mm^3^ or serum creatinine <200 μmol/L (2.27 mg/dL); score of 0: platelet count ≥30 × 10^3^/mm^3^ and serum creatinine ≥200 μmol/L (2.27 mg/dL).^20^

b Patients at high risk of early death of iTTP were defined by the French severity score ≥3 (cerebral involvement: yes = 1/no = 0, LDH: >10 × ULN = 1/≤10 × ULN = 0, age: >60 years = 2/>40 and ≤ 60 years = 1/≤40 years = 0)^7^. Continuous variables are provided as median (Q1–Q3); qualitative variables are provided as percentage of patients in the respective treatment group with valid data. P-value was considered significant when <0.05.

### Primary outcome

**Fig. 2:**
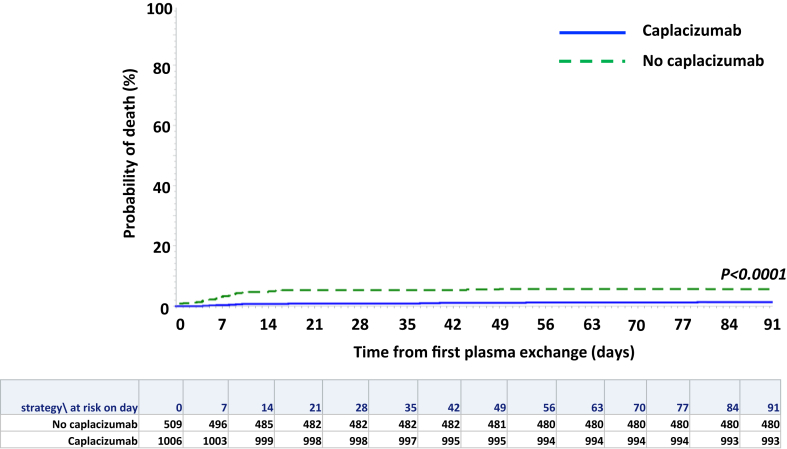
Cumulative probability of death after first therapeutic plasma exchange (TPE) up to 3 months in patients of the caplacizumab group (blue line) versus patients of the control group (green dashed line).

The 3-month survival post-first TPE in the caplacizumab group, regardless of time of caplacizumab initiation, was 98.5% and 94.0% in the control group (Fig. 2; P < 0.0001). Hence, 3-month mortality was 4.2-fold higher in the control group as compared to the caplacizumab group (95% CI OR: 2.22–7.7, P < 0.0001), and the estimated number of patients needed to treat with caplacizumab to prevent one death was 22. Death in the caplacizumab group (N = 15/1015) was considered as directly related to uncontrolled iTTP in eight cases (53%); in four others, death resulted from iTTP-related comorbidities, including one case of fatal intracerebral hemorrhage late in the management, while iTTP was in clinical response; in the three remaining patients, death was not related to iTTP (Supplemental Table S3). Among these 15 patients, 12 (80%) had received caplacizumab within three days following first TPE. In the control group, death was directly related to an uncontrolled iTTP in all 26 patients with available data (Supplemental Table S4).

Rituximab was used in patients of the caplacizumab group more often than in the control cohort (90.5% and 71%, respectively, P < 0.0001) (Table 1). Although the role of rituximab in the acute phase of iTTP is primarily to achieve ADAMTS13 activity recovery that typically begins within two to three weeks,^6^^,^^21^ we tested whether rituximab had an impact on mortality. By comparing patients according to therapeutic groups, we found that 3-month survival remained better in patients in the caplacizumab group as compared to controls, whether they received rituximab (98.5% versus 95.6%, respectively, P = 0.001) or not (97.8% versus 90.5%, respectively, P = 0.03). Moreover, we found that within the caplacizumab group, 3-month survival was similar between patients who received rituximab and those who did not (Supplemental Figure S1), supporting the view that caplacizumab, through an immediate role on inhibiting platelet clumping, improves iTTP survival independent of the use of rituximab.

The prevalence of elderly patients (>60 years^22^) at diagnosis was comparable between both groups (P = 0.14) (Table 1). However, among non-survivors, the proportion of elderly patients was higher in the caplacizumab group than in the control group (11/15 [73%] and 10/30 [33%], respectively, P = 0.025) (Supplemental Table S5); among all non-survivors, median age was 65.5 years [Q1–Q3, 58.7–70.2] in the caplacizumab group and 54 years [Q1–Q3, 43.2–63.8] in controls (P = 0.01). Death occurred in controls 7 days after the first TPE (Q1–Q3, 4–9) and hence earlier than in the caplacizumab group (11 days [Q1–Q3, 9–33] from first TPE, P = 0.01). By analyzing reported prognostic factors associated with death, age remained the prognostic factor most associated with 3-month survival, regardless of therapeutic regimens (Supplemental Table S5). To further demonstrate the robustness of our findings to potential confounding factors, we assessed whether caplacizumab was independently associated 3-month survival. By comparing survivors to non-survivors, we found that age, increased troponin level at baseline, and the use of caplacizumab, corticosteroids and rituximab were all associated with 3-month survival (Supplemental Table S6). By multivariate analysis, only age and the use of caplacizumab remained associated with survival (Supplemental Table S7).

### Key secondary outcomes

**Fig. 3:**
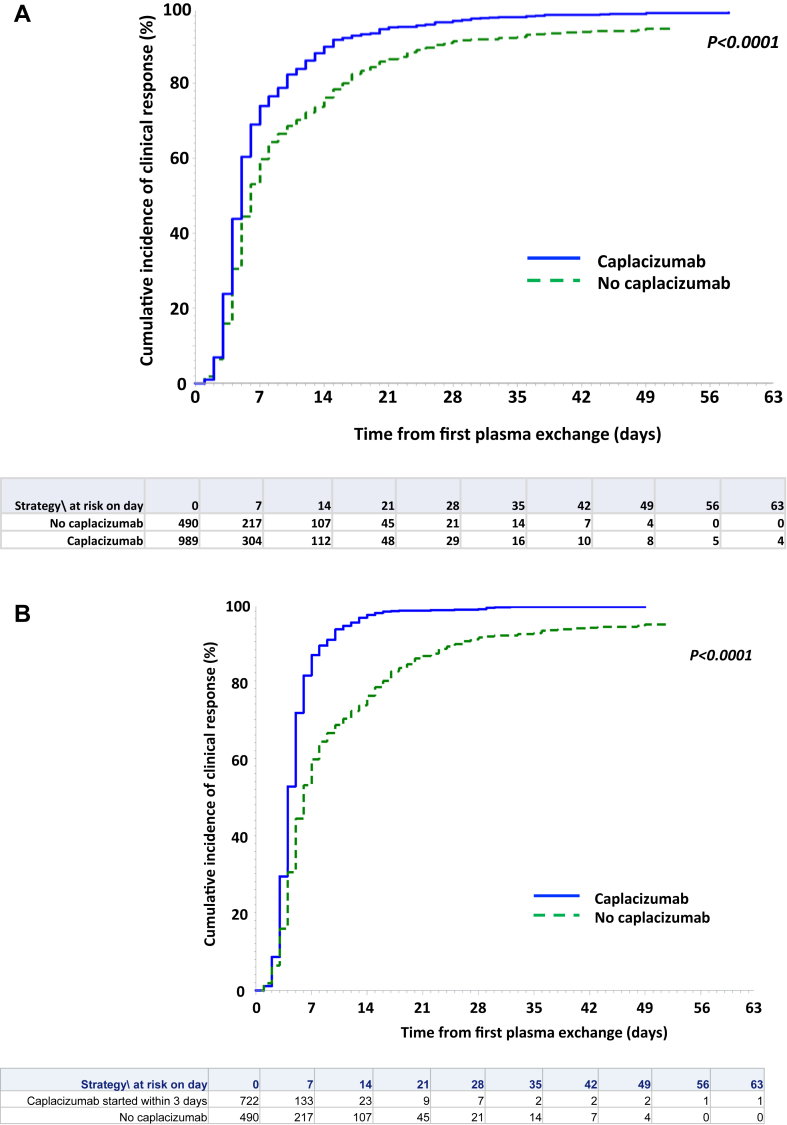
Cumulative incidence of clinical response after first therapeutic plasma exchange (TPE) (**A**) in patients treated with caplacizumab (blue line) versus patients of the control group (green dashed line), and (**B**) in patients treated with caplacizumab early (i.e., within three days from first TPE; blue line) versus patients of the control group (green dashed line).

Patients receiving caplacizumab experienced less frequent refractoriness than controls (P < 0.0001) (Table 2). Median time to clinical response in the caplacizumab group was shorter than in controls (5 days [Q1–Q3, 4–8] versus 6 days [4–12] from first TPE, respectively, P < 0.0001) (Table 2) (Fig. 3A; Log–Rank test, P < 0.0001). By considering patients who received caplacizumab according to the recommendation, i.e., early in the management, median time to clinical response was 4 days (Q1–Q3, 3–6) from first TPE, and the mean time to clinical response was half the time compared to control group (5.7 ± 5.6 days versus 11 ± 9.4 days from first TPE, respectively) (P < 0.0001) (Fig. 3B; Log–Rank test, P < 0.0001). In patients with delayed caplacizumab initiation, clinical response was obtained after a median of 4 days (Q1–Q3, 2–8) from caplacizumab initiation, which covers the range observed for patients who initiated caplacizumab according to recommendations (P = 0.85).

**Table 2:** Key secondary outcomes according to therapeutic groups.

	Caplacizumab group (N = 1015)	Control group (N = 510)	P-value
Clinical response	99%	94%	<0.0001
Refractoriness	1%	10.1%	<0.0001
Time to death from first TPE (days)	11 (9–33)	7 (4–9)	0.01
Time to clinical response (days)	5 (4–8)	6 (4–12)	<0.0001
Number of TPE to achieve clinical response	5 (4–8)	7 (4–16)	<0.0001
Exacerbation rate	4%^a^	32%	<0.0001
	(N = 867)	(N = 320)	

Time to ADAMTS13 activity ≥20% from first TPE (days)	29 (17–50)	31 (17–65)	0.07
Within 28 days (n)	430 (49.6%)	150 (47%)	0.47
Within 29–56 days (n)	260 (30%)	73 (23%)	<0.05
>56 days (n)	177 (20.4%)	97 (30%)	<0.001

Abbreviations: TPE, therapeutic plasma exchange; ADAMTS13, A Disintegrin And Metalloproteinase with ThromboSpondin-1 motifs; member 13.

a While on caplacizumab treatment. Continuous variables are provided as median (Q1–Q3); qualitative variables are provided as percentage of patients in the respective treatment group with valid data. P-value was considered significant when <0.05.

**Fig. 4:**
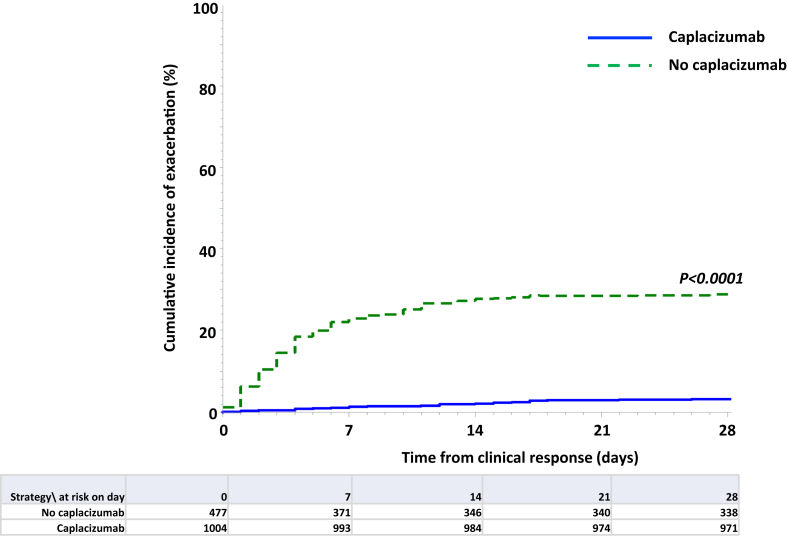
Cumulative incidence of exacerbation after clinical response in patients of the caplacizumab group (blue line) versus patients of the control group (green dashed line).

Following clinical response, exacerbations were less frequent in caplacizumab-treated patients than among patients of the control group, with an OR of 0.08 (CI 95%: 0.06–0.12) (Table 2; Fig. 4; P < 0.0001). There was a trend towards a more rapid recovery of ADAMTS13 activity ≥20% from first TPE in patients of the caplacizumab group (P = 0.07); moreover, the percentage of patients with a strongly delayed ADAMTS13 recovery (>56 days) was lower in the caplacizumab group where rituximab was more systematically used, versus the control group (P < 0.001) (Table 2). Among 1007 patients who achieved clinical response, 76 exacerbations occurred (7.5%) following the interruption of caplacizumab (generally because the maximal duration of recommended treatment, i.e., 30-day post-last TPE + 28 additional days^8^ had been reached, or because caplacizumab was administered every other day^23^), while ADAMTS13 was still undetectable. In most cases, exacerbation was characterized by only thrombocytopenia, with favorable outcomes achieved when caplacizumab administration was resumed with daily dosing. By contrast, no patients of the caplacizumab group that had recovered ADAMTS13 activity to ≥20% before stopping the treatment suffered an iTTP exacerbation.

The estimated number of patients needed to treat with caplacizumab to prevent one context of refractoriness and one exacerbation were 11 and 3.6, respectively.

### Adverse events

Two-hundred and fifteen (21%) patients experienced 239 caplacizumab-related adverse events. They consisted of major bleeding in 24 patients (2.4%); in 17 patients, caplacizumab was discontinued; in 5 others, it was suspended 1–11 days and subsequently resumed at the same dose or half-dose (2 and 3 cases each) (data were missing for two patients). Intracerebral hemorrhage occurred in three patients (Supplemental Table S8). Clinically relevant non-major bleeding (N = 38 patients; 3.7%) and non-clinically relevant non-major bleeding (N = 116 patients, 11.4%) were also observed (Table 3). In most cases, adverse events were manageable and caplacizumab was continued, although one patient with pneumopathy-associated hemoptysis required infusions of factor VIII and VWF concentrates. Thirty-seven patients (3.6%) developed inflammatory reaction consisting in a swelling at the injection sites of caplacizumab that occurred typically toward the end of the treatment course; however, no premature interruption of the treatment was needed (Table 3). Patients with major bleeding were older than those with non-major bleeding or no adverse event (57 [Q1–Q3, 47–63.5] versus 45 [Q1–Q3, 33–58] years old, respectively, P = 0.006).

**Table 3:** Caplacizumab-related adverse events.

	Number of patients with adverse events (%)	Description^b^
Major bleeding	24 (2.4)^a^	- Massive digestive bleeding (N = 10); - Abundant menorrhagia with a decrease in hemoglobin level of at least 2 g/dL (N = 3); - Massive bleeding at the catheter site insertion (N = 2); - Hemoptysis with underlying pneumopathy (including one SARS-COV-2 infection) (N = 2); - Intracerebral hemorrhage (N = 3), including one hemorrhagic transformation of stroke; - Severe epistaxis requiring hemostatic surgery (N = 1); - Periorbital hemorrhage with blurred vision (N = 1);
Clinically relevant non major bleeding	38 (3.7)	- Epistaxis (N = 12); - Bleeding at the femoral catheter site insertion (N = 8); - Metrorrhagia (N = 7); - Macroscopic gastrointestinal hemorrhage (N = 4); - Gingival bleeding (N = 4); - Hematoma (N = 2); - Bruises (N = 2); - Hematuria (N = 1); - Bleeding following an invasive procedure (tracheal intubation) (N = 1); - Accidental cut (N = 1);
Non clinically relevant non major bleeding	116 (11.4)	- Epistaxis (N = 46); - Gingival bleeding (N = 29); - Bruises (N = 19); - Meno-metrorrhagia (N = 18); - Hematoma (N = 6); - Digestive bleeding (N = 5); - Bleeding at the catheter site insertion (N = 5); - Conjunctival hemorrhage (N = 4); - Hemoptoic sputum (N = 2); - Hematuria (N = 1); - Bleeding following tracheal intubation (N = 1);
Inflammatory reaction	37 (3.6)	Inflammatory swelling at the injection site, especially at the end of the treatment course.

a Data are missing for two patients.

b 24 patients experienced two adverse events. SARS-COV-2: Severe Acute Respiratory Syndrome-Coronavirus-2.

## Discussion

The survival of patients with iTTP during the acute phase remained broadly unchanged since the systematic use of TPE.^3^^,^^4^ Although integrated analyses from pivotal trials as well as meta-analyses^10^^,^^11^ suggested that survival in iTTP could be improved with caplacizumab, controversies remained.^12^^,^^13^^,^^24^ We report here the largest international series of caplacizumab-treated patients and provide evidence that caplacizumab, added to daily TPE and immunosuppression, improves the outcome of acute iTTP by preventing refractoriness and exacerbations, with a 4-fold reduction of 3-month mortality. The reduced mortality in the caplacizumab group was not due to less severe iTTP episodes as the severity of the disease in this group was comparable to controls in terms of clinical presentations, though both cohorts are representative of the whole iTTP population. The number of patients needed to treat with caplacizumab to prevent any unfavorable outcome was particularly low (i.e., 3–4 patients), providing further arguments that caplacizumab is efficient in controlling acute iTTP.

A key observation here is that nearly all deaths observed in the control group occurred early during the management, typically within days from diagnosis and systematically in a context of uncontrolled iTTP.^7^ Moreover, apart from age, we could not identify clear predictors of mortality, especially in the caplacizumab group. Hence, a delayed introduction of caplacizumab during the management may expose patients to a higher risk of unpredictable refractoriness and exacerbation, which may precede death,^11^ arguing for a systematic use of caplacizumab early in the management, typically as soon as the diagnosis of iTTP and the decision to start TPE are made, to prevent the formation of deleterious microthrombi and prevent adverse outcomes.^25^ In this context, we also found that the early use of caplacizumab could substantially alleviate the burden of care, with a decrease in time to clinical response and in the number of needed TPE sessions when compared to controls. We could also confirm the efficacy of caplacizumab in patients with an uncontrolled disease under only TPE, as they systematically achieved a prompt clinical response after caplacizumab was initiated as a salvage therapy.^8^

Age, neurologic involvement, very high LDH levels, and raised cardiac troponin were historically reported as risk factors associated with death in iTTP.^7^^,^^26^^,^^27^ In our cohort, age remained strongly associated with a worse outcome despite the use of caplacizumab, although iTTP survival in the elderly was improved with caplacizumab compared to controls.^22^ Troponin level was less prognostic with the use of caplacizumab; similarly, LDH level and neurologic involvement had less prognostic value, although the French severity score remained discriminant for prognosis mostly because of age. These findings suggest that caplacizumab could at least in part circumvent the historical factors of poor prognosis in iTTP at the acute phase, clearly supporting its use. Of particular interest, we observed that non-survivors in the caplacizumab group were more frequently elderly patients who succumbed later from underlying comorbidities and from complications related to hospitalization while iTTP was in clinical response. This hitherto unreported observation suggests that caplacizumab has changed the pattern of death in iTTP that now predominantly occurs in patients with frailty and comorbidities, which could represent confounders for increased mortality in the elderly. These statements contrast with historic reports where non-survivors were typically younger patients experiencing multiorgan failure in a context of rapidly progressive refractory disease.^7^ New strategies to better control age-related comorbidities and prevent post-iTTP complications are needed with caplacizumab-containing regimens to further improve the prognosis of the disease.

The efficacy of caplacizumab in the treatment of iTTP was also documented in recent years by the successful treatment of patients with caplacizumab-based, TPE-free regimens.^28^^,^^29^ Although under current investigation through formal clinical trials (Clinicaltrials.gov, #NCT05468320), this strategy provides further convincing evidence that caplacizumab, in addition to immunosuppression, is efficient in the control of iTTP, and opens an additional perspective to alter future regimens.

Adverse events were in line with those in previous reports,^8^^,^^9^^,^^11^ and were usually manageable. We noted, however, that gastrointestinal bleeding was prevalent among severe events, followed by meno-metrorrhagia, intracerebral hemorrhage, and severe central venous catheter insertion site bleeding. Expectedly, severe bleeding events were more frequently observed in the elderly population, where one can assume that comorbidities and polypharmacy including anti-platelet agents and anticoagulants are more prevalent. In these patients exposed to a higher bleeding risk, the use of caplacizumab remains controversial and deserves further investigation. Intracerebral hemorrhage resulting from hemorrhagic transformation of ischemic stroke was reported in desperate cases with multiple cerebral infarcts, especially when caplacizumab initiation is delayed.^14^ Intracerebral hemorrhage remains a potentially serious complication in iTTP patients, that could be facilitated by caplacizumab; hence, a rigorous evaluation of risk factors for intracerebral hemorrhage at baseline should be carried out and the indication for caplacizumab made on a case by case basis.^30^

The cost of caplacizumab represents one of the main limitations in its use, although its possible cost-effectiveness was recently suggested.^31^ In this context, it is crucial to hasten ADAMTS13 activity improvement to stop caplacizumab treatment as soon as safely possible. Here, half of patients were slow responders as they achieved a protective level of ADAMTS13 activity beyond 28-day post-TPE, with 20% of patients requiring more than two months of caplacizumab post-TPE in the more extreme cases. Surrogate markers are therefore urgently needed to identify earlier these patients less likely to recover the ADAMTS13 activity in the first 30 days^32^ to allow for increased intensity or additional rational alteration of the immunosuppressive regimen. In this work, we found apparently no clear difference in time to ADAMTS13 improvement between patients treated with caplacizumab and those of the control group, which contrasts with a recent report.^33^ Therefore, whether time to ADAMTS13 improvement is prolonged in the era of caplacizumab remains a matter of controversy and deserves further investigations.^34^

The main limitation of our work is its retrospective and multicentric design with the use of a historical control cohort, providing possible bias regarding recruitment and data homogeneity; especially, patients with African and West Indies ancestry were more represented in the caplacizumab group, possibly as a consequence of country-recruitment bias between therapeutic groups. Moreover, the recruitment of patients involved only experienced teams, as reflected by the low death rate in both therapeutic groups; hence, our results may only apply to participating centers. However, the recruitment of patients by experienced teams with expertise in the management of iTTP with caplacizumab guaranteed homogeneous and comparable cohorts of patients, and practitioners applied the standardized definitions for outcomes. Moreover, we considered here only cases managed according to the most recent standards,^18^ as well as controls selected within a 3-year restricted period before the systematic caplacizumab use to guarantee therapeutic homogeneity. Second, the use of rituximab was variable between teams and its use was more systematic in the caplacizumab group, reflecting a wider adoption of rituximab in the more recent patients; however, virtually all patients in the caplacizumab group achieved clinical response within 8 days, which is by far faster than the previous two to three weeks onset of ADAMTS13 response observed with rituximab.^5^^,^^6^ Therefore, rituximab would not be expected to have significant impact on prognosis at the acute phase of the disease, but instead later by hastening ADAMTS13 activity improvement allowing caplacizumab discontinuation; our results thus provide evidence that caplacizumab's efficacy on survival is obtained regardless of the use of rituximab, and that both agents have complementary actions by addressing distinct aspects of iTTP pathophysiology. Lastly, studies with larger numbers of patients should identify in more detail prognostic factors taking into account all confounding factors.

In conclusion, this international academic effort on such a rare disease provides additional evidence that caplacizumab improves iTTP prognosis in the acute phase by achieving impressive survival rates of up to 98.5% while decreasing the TPE-related burden of care. Efforts are now required to evaluate TPE-free^29^ or -alleviated regimens, but also more efficient immunomodulation regimens in slow responders to standard immunosuppression to offset the cost burden of prolonged use of caplacizumab.

## Contributors

P. Coppo initiated and designed the study; enrolled patients and collected, accessed and verified the clinical data; analyzed the data and wrote the first version of the manuscript; M. Bubenheim accessed and verified the clinical data and performed the statistical analyses; S. Cataland and M. Scully initiated and designed the study; enrolled patients and collected clinical data; analyzed the data and extensively edited the manuscript; B. Lämmle initiated and designed the study, analyzed the data and extensively edited the manuscript; Y. Benhamou, L. Völker, P. Brinkkötter, L. Kühne, P. Knöbl, M.E. Mingot-Castellano, C. Pascual-Izquierdo, J. de la Rubia, J. del Rio Garma, S. Chaturvedi, C. Masias, M. Mazepa, X.L. Zheng, G. Sinkovits, M. Réti, C.J. Patriquin, K. Pavenski, T. Boechat, J. Farias, E. Flavio Oliveira Ribeiro, M. Larissa Lobo de Andrade, K. Sakai, M. Matsumoto, P. Agosti, I. Mancini, F. Peyvandi, M. Stubbs and A. Hmaid enrolled patients and collected clinical data; A. Veyradier and B. Joly performed ADAMTS13 explorations for French patients; R. Bouzid collected the data for French patients and accessed and verified all the clinical data; she prepared the datafile for statistical analyses and the flow-chart figure; E. Gavriilaki provided comprehensive and consistent patients data after the enrollment period. All authors read and approved the final version of the manuscript, and substantially improved the manuscript. All authors had full access to all the data in the study and accepted responsibility to submit for publication.

## Data sharing statement

Data on which this article is based can be made available upon reasonable request to the corresponding author.

## Declaration of interests

P. Coppo is member of the Clinical Advisory Board for Alexion, Sanofi-Genzyme and Takeda. L. Kühne received consulting fees from Alexion and research funding from Sanofi-Genzyme. L.A. Völker received research funding and consulting fees from Alexion, AstraZeneca, Bayer, GC Biopharm and Sanofi-Genzyme, GC Biopharm. P.T. Brinkkötter received speaker honoraria and consultant fees from AstraZeneca, Alexion, Bayer, Boehringer-Ingelheim, Novartis, Roche, Sanofi-Genzyme, Travere, Vifor CSL and participated in advisory boards for Alexion, Sanofi-Genzyme, Novartis, Travere, Takeda, Vifor CSL and Bayer. He declares research funding from the German Research Foundation BR-2955/8 and Sanofi-Genzyme. P. Knöbl is member of the Clinical Advisory Boards for Sanofi and Takeda. T. Boechat has participated to advisory boards for Sanofi-Genzyme. Y. Benhamou, B. Joly and A. Veyradier have participated to Advisory boards for Sanofi-Genzyme and Takeda. M.E. Mingot-Castellano is member of the Clinical Advisory Board for Alexion, Werfen, Sanofi-Genzyme and Takeda, and grants from the 3 companies. X. Long Zheng is a consultant for Alexion, Apollo, GC Biopharma, Sanofi-Genzyme, Stago, and Takeda. X.L.Z. is also the co-founder of Clotsolution; he also received educational grant funding to support 2024 ISTH congress satellite symposium for platelets and hemostasis, Bangkok, Thailand, and grants unrelated to the present study, from NHLBI (HL157975-01A1 and HL164016-01A1) and Answering T.T.P. Foundation. Marienn Reti received congress support by Sanofi-Genzyme (EHA, 2024) and participated to the iTTP European and International Region Medical Advisory Board (2023). C.J. Patriquin has receive consultancy honoraria from Alexion, BioCryst, Novartis, Roche, Sanofi-Genzyme, Sobi, and Takeda, and speaking honoraria from Alexion, Amgen, and Sobi. K. Pavenski provided unpaid consultancy to Sanofi-Genzyme and Takeda; served as site PI in industry trials of Sanofi-Genzyme, Takeda, Roche and SOBI. She received honoraria from France foundation for travel and presentations and honoraria from Octapharma for travel. K. Sakai received lecture fees from Sanofi-Genzyme. M. Matsumoto has provided consultancy services for Takeda, Alexion and Sanofi-Genzyme, and has received speaker fees for Takeda, Alexion, Asahikasei Pharma, and Sanofi-Genzyme and has received research funding from Alexion, Chugai Pharmaceutical, Asahikasei Pharma, and Sanofi-Genzyme. P. Agosti received honoraria for participating as a speaker at educational meetings organized by Sanofi-Genzyme. I. Mancini received honoraria for participating as a speaker at educational meetings organized by Werfen and Sanofi-Genzyme. F. Peyvandi received honoraria for participating in symposia and educational events for Takeda/Spark and in advisory boards for Biomarin, Roche, Sanofi-Genzyme, Sobi, and CSL Behring. S. Cataland is member of the Clinical Advisory Board for Sanofi-Genzyme and Takeda. M. Scully is member of the Clinical Advisory Board for Alexion, Sanofi-Genzyme and Takeda. B. Lämmle is chairman of data monitoring committees of studies investigating recombinant ADAMTS13 for the treatment of congenital and acquired TTP (Takeda), chairman of a steering committee analyzing global impact of congenital TTP (Takeda), and chairman of the data monitoring committee of Mayari study (investigating caplacizumab for the treatment of autoimmune TTP without plasma exchange (Sanofi-Genzyme)). The other authors do not have any conflict of interest to declare.

## Appendix A Supplementary data

Appendix-Study Team Members

Supplemental Figure S1

Supplemental Material Capla 1000+280225

Supplemental File 1 Capla 1000+ dataset
