# Supplementary material for: Caplacizumab use in immune-mediated thrombotic thrombocytopenic purpura: an international multicentre retrospective Cohort study (The Capla 1000+ project)
Source: eClinicalMedicine. 2025 Mar 30;82:103168. doi: 10.1016/j.eclinm.2025.103168 (PMC11997362; doi:10.1016/j.eclinm.2025.103168)
Supplement: Appendix-Study Team Members [file mmc1.docx]

**Appendix**

**Members of the French team (Reference Center for Thrombotic Microangiopathies [CNR-MAT]):**

Augusto Jean-François (Service de Néphrologie, dialyse et transplantation ; CHU Larrey, Angers); Azoulay Elie (Service de Réanimation Médicale, Hôpital Saint-Louis, Paris); Barbay Virginie (Laboratoire d’Hématologie, CHU Charles Nicolle, Rouen); Benhamou Ygal (Service de Médecine Interne, CHU Charles Nicolle, Rouen); Cador-Rousseau Bérengère (Service de Néphrologie, Hôpital Pontchaillou, Rennes); Charvet-Rumpler Anne (Service d’Hématologie, CHU de Dijon) ; Chauveau Dominique, Ribes Davis (Service de Néphrologie et Immunologie Clinique, CHU Rangueil, Toulouse); Choukroun Gabriel (Service de Néphrologie, Hôpital Sud, Amiens); Coindre Jean-Philippe (Service de Néphrologie, CH Le Mans); Coppo Paul (Service d’Hématologie, Hôpital Saint-Antoine, Paris); Delmas Yahsou (Service de Néphrologie, CHU de Bordeaux, Bordeaux); Kwon Theresa (Service de Néphrologie Pédiatrique, Hôpital Robert Debré, Paris); Salanoubat Célia (Service d’Hématologie, Hôpital Sud-Francilien, Corbeil-Essonnes); Dossier Antoine (Service de Néphrologie, Hôpital Bichat, Paris); Fain Olivier (Service de Médecine Interne, Hôpital Saint-Antoine, Paris); Ville Simon (Service de Néphrologie, CHU Hôtel-Dieu, Nantes) ; Frémeaux-Bacchi Véronique (Laboratoire d’Immunologie, Hôpital Européen Georges Pompidou, Paris); Galicier Lionel (Service d’Immunopathologie, Hôpital Saint-Louis, Paris); Grangé Steven (Service de Réanimation Médicale, CHU Charles Nicolle, Rouen) ; Guidet Bertrand (Service de Réanimation Médicale, Hôpital Saint-Antoine, Paris); Halimi Jean-Michel (Service de Néphrologie Pédiatrique, Hôpital Bretonneau, Tours); Hamidou Mohamed, Neel Antoine (Service de Médecine Interne, Hôtel-Dieu, Nantes); Fornecker Luc-Matthieu (service d’Oncologie et d’Hématologie, Hôpital de Hautepierre, Strasbourg); Hié Miguel (Service de Médecine Interne, Groupe Hospitalier Pitié-Salpétrière, Paris) ; Jacobs Frédéric (Service de Réanimation Médicale, Hôpital Antoine Béclère, Clamart); Joly-Laffargue Bérangère (Service d’Hématologie Biologique, Hôpital Lariboisière, Paris) ; Kanouni Tarik (Unité d’Hémaphrèse, Service d’Hématologie, CHU de Montpellier) ; Kaplanski Gilles (Service de Médecine Interne, Hôpital la Conception, Marseille) ; Rieu Claire (Hôpital d’Estaing, Service de Médecine Interne, Clermont-Ferrand); Le Guern Véronique (Unité d’Hémaphérèse, Service de Médecine Interne, Hôpital Cochin, Paris) ; Moulin Bruno (Service de Néphrologie, Hôpital Civil, Strasbourg); Rebibou Jean-Michel (Service de Néphrologie, CHU de Dijon); Ojeda Uribe Mario (Service d’Hématologie, Hôpital Emile Muller, Mulhouse); Parquet Nathalie (Unité de Clinique Transfusionnelle, Hôpital Cochin, Paris); Pène Frédéric (Service de Réanimation Médicale, Hôpital Cochin, Paris) ; Perez Pierre (Service de Réanimation polyvalente, CHU de Nancy) ; Poullin Pascale (Service d’hémaphérèse et d’autotransfusion, Hôpital la Conception, Marseille); Marie Manon (Service de Néphrologie, CHU Lyon-Sud, Lyon); Presne Claire (Service de Néphrologie, Hôpital Nord, Amiens); Provôt François (Service de Néphrologie, Hôpital Albert Calmette, Lille); Mesnard Laurent (Urgences Néphrologiques et Transplantation Rénale, Hôpital Tenon, Paris); Saheb Samir (Unité d’Hémaphérèse, Hôpital la Pitié-Salpétrière, Paris) ; Seguin Amélie (Service de Réanimation Médicale, CHU Hôtel-Dieu, Nantes) ; Servais Aude (Service de Néphrologie, CHU Necker-Enfants Malades) ; Stépanian Alain (Laboratoire d’Hématologie, Hôpital Lapeyronie, Montpellier); Veyradier Agnès (Service d’Hématologie Biologique, Hôpital Lariboisière, Paris); Wynckel Alain (Service de Néphrologie, Hôpital Maison Blanche, Reims); Zunic Patricia (Service d’Hématologie, Groupe Hospitalier Sud-Réunion, la Réunion).

**Members of the German team (REACT-2020 study group):**

Anja Mühlfeld (Uniklinik RWTH Aachen, Department of Medicine, Division of Nephrology, Aachen), Matthias Hermann (Medizinische Klinik V, Hämatologie/Onkologie, Sozialstiftung Bamberg, Klinikum am Bruderwald, Bamberg), Markus Bieringer (Department of Cardiology and Nephrology, Helios Klinik Berlin-Buch, Berlin), Wolfram J. Jabs (Department of Nephrology, Vivantes Klinikum im Friedrichshain, Berlin), Kristin Sauerland (Klinik für Innere Medizin, Hämatologie/Onkologie, Stammzelltransplantation und Palliativmedizin, Johannesstift, Bielefeld), Jörn Bramstedt (Medizinische Klinik II Sektion Nephrologie, Klinikum Bremerhaven Reinkenheide, Bremerhaven), Evelyn Seelow (Department of Nephrology and Intensive Care Medicine, Charité - Universitätsmedizin Berlin, Berlin), Adrian Schreiber (Department of Nephrology and Intensive Care Medicine, Charité - Universitätsmedizin Berlin, Berlin), Markus Tölle (Department of Nephrology and Intensive Care Medicine, Berlin Institute of Health, Berlin), Anke Mogner (Department of Internal Medicine III, Klinikum Chemnitz GmbH, Chemnitz), Regina Herbst (Department of Internal Medicine III, Klinikum Chemnitz GmbH, Chemnitz), Fedai Özcan (Department of Nephrology, Klinikum Dortmund, Universität Witten Herdecke), Sebastian A. Potthoff (University Hospital Düsseldorf, Department of Nephrology, Medical Faculty, Heinrich-Heine University, Düsseldorf), Ulrich P. Hinkel (Klinik für Nephrologie, Zentralklinik Bad Berka GmbH, Bad Berka), Anja Gäckler (Department of Nephrology, University Hospital Essen, University of Duisburg-Essen, Essen), Wolfgang Miesbach (Department of Hemostaseology-Hemophilia Center, University Hospital Frankfurt, Frankfurt), Johanna Schneider (Department of Nephrology and Primary Care, Medical Center–University of Freiburg, Faculty of Medicine, University of Freiburg), Martin Bommer (Department of Internal Medicine, Hematology, Oncology, Palliative Care, and Infectious Diseases, Alb-Fils-Kliniken, Göppingen), Silke Markau (Department of Internal Medicine II, Martin Luther University Halle-Wittenberg, Halle), Matthias Girndt (Department of Internal Medicine II, Martin Luther University Halle-Wittenberg, Halle), Victor Walendy (Department of Internal Medicine II, Martin Luther University Halle-Wittenberg, Halle), Tilman Schmidt (Section of Nephrology, Clinic and Policlinic of Internal Medicine A, University Medicine Greifswald, Ferdinand-Sauerbruch-Straße, 17475 Greifswald), Jessica Kaufeld (Department of Nephrology and Hypertension, Medical School Hannover, Hannover), Jan Menne (KRH Klinikum Mitte – Location Siloah, Hannover), Felix S. Seibert (Medical Department I, Marien Hospital Herne, Ruhr-University Bochum), Timm Westhoff (Medical Department I, Marien Hospital Herne, Ruhr-University Bochum), Helmut Felten (Department of General Internal Medicine, Nephrology, Rheumatology, and Pneumology, Karlsruhe General Hospital, Karlsruhe), Martin Hausberg (Department of General Internal Medicine, Nephrology, Rheumatology, and Pneumology, Karlsruhe General Hospital, Karlsruhe), Benedikt Kolbrink (Department of Nephrology and Hypertension, University Hospital Schleswig-Holstein, Kiel), Kevin Schulte (Department of Nephrology and Hypertension, University Hospital Schleswig-Holstein, Kiel), Heike Bruck (Medical Clinic III, Helios Hospital Krefeld, Krefeld), Maximilian Roeder (Section of Nephrology, Medical Clinic I, Klinikum Landshut, Landshut), Ralph Wendt (Department of Infectious Diseases/Tropical Medicine, Nephrology/KfH Renal Unit and Rheumatology, St. Georg Hospital Leipzig), Gilles Klein (Centre Hospitalier Emile Mayrisch, Esch-sur-Alzette, Luxembourg), Charis von Auer (Department of Hematology, Oncology, and Pneumology, University Medical Center of the Johannes Gutenberg University, Mainz, Rheinland-Pfalz), Ana Harth (Department of Nephrology, Transplantation, and Medical Intensive Care, University Witten/Herdecke, Medical Centre Cologne-Merheim, Cologne), Jörg Radermacher (Center for Internal Medicine/Nephrology, Johannes Wesling Klinikum Minden, University Hospital of the Ruhr-University of Bochum, Herne), Ulf Schönermarck (Nephrology Division, Department of Medicine IV, University Hospital, LMU Munich, Munich), Anke von Bergwelt-Baildon (Nephrology Division, Department of Medicine IV, University Hospital, LMU Munich, Munich), Marcus Brand (Department of Internal Medicine D, University of Münster, Muenster), Tobias J. Müller (Department of Neurology, University Hospital Ruppin-Brandenburg, Brandenburg Medical School, Neuruppin), Saban Elitok (Department of Nephrology and Endocrinology/Diabetology Potsdam, Klinikum Ernst von Bergmann, Potsdam), Alexander Gawlik (Department of Nephrology and Endocrinology/Diabetology Potsdam, Klinikum Ernst von Bergmann, Potsdam), Kristin Klemm (Department of Nephrology and Endocrinology/Diabetology Potsdam, Klinikum Ernst von Bergmann, Potsdam), Vedat Schwenger (Department of Nephrology, Klinikum Stuttgart, Stuttgart), Jens Gerth (Department of Internal Medicine II,Heinrich Braun Klinikum Zwickau, Zwickau), Lena Schulte-Kemna (Section of Nephrology, University Hospital, Ulm), Christian Pfrepper (Medical ICU, University of Leipzig Medical Center, Leipzig), Johannes Ruhe (Department of Internal Medicine III, Nephrology, University Hospital Jena - Friedrich Schiller University, Jena), Dennis A. Eichenauer (Department I of Internal Medicine, Center for Integrated Oncology, Aachen Bonn Cologne Dusseldorf, University of Cologne, Cologne).

**Members of the UK team:**

Louisa Keogh, Sobia Sharif (University College London Hospitals NHS Foundation Trust, London) ; Rennick Gregory , Joost Vanveen (Sheffield haemophilia and thrombosis centre); Julie Kenny, Joannes Hermans (Nottingham University Hospitals); Jayna Mistry , Will Lester, Pamela Green  (University Hospitals Birmingham); Joanne Bell, Tina Dutt (Liverpool University Hospitals); Will Thomas, Matthew Carter (Cambridge University Hospitals); Amanda Clarke (University Hospitals Bristol) ; Sayma Raza-Burton, Michael Desborough (Oxford University Hospitals); Sharon Bluett , Samya Gwen Obaji (University Hospital of Wales (Cardiff)); and collaborators of the UK TTP registry.

**Members of the Spanish team:**

FERNÁNDEZ SÁNCHEZ DE MORA Maria Carmen (Hospital Universitario Reina Sofía, CORDOBA) ; MORENO BELTRÁN María Esperanza (Hospital Costa del Sol, MARBELLA) ; TALLÓN Inmaculada (Hospital VIRGEN MACARENA, SEVILLA) ; GONZALEZ RODRIGUEZ Victoria Paz (HOSPITAL UNIVERSITARIO MIGUEL SERVET, ZARAGOZA) ; MORENO CHULILLA José Antonio (HOSPITAL CLINICO UNIVERSITARIO LOZANO BLESA, ZARAGOZA) ; GARCÍA GALA José Maria (HOSPITAL UNIVERSITARIO CENTRAL DE ASTURIAS, OVIEDO) ; GUERRA DOMÍNGUEZ Luisa (HOSPITAL DE GRAN CANARIA DR. NEGRÍN, LAS PALMAS) ; OLIVA HERNANDEZ Ana Yurena (HOSPITAL UNIVERSITARIO NUESTRA SEÑORA DE LA CANDELARIA, TENERIFE) ; AMUNARRIZ Cristina (HOSPITAL UNIVERSITARIO MARQUES DE VALDECILLA, SANTANDER) ; MORALES SANZ Maria Dolores (HOSPITAL DE GUADALAJARA, GUADALAJARA) ; CAMPUZANO Verónica (HOSPITAL GENERAL UNIVERSITARIO DE BURGOS, BURGOS) ; VIDAN ESTEVEZ Julia (COMPLEJO ASISTENCIAL UNIVERSITARIO DE LEON, LEON) ; CID VIDAL Joan (HOSPITAL CLÍNIC, BARCELONA) ; GARCIA ARROBA PEINADO José (HOSPITAL JOAN XXIII, TARRAGONA) ; SIMONA Gabriela (HOSPITAL DEL MAR, BARCELONA) ; JIMENEZ Maria Moraima (HOSPITAL VALL D´HEBRON, BARCELONA) ; SOLANICH MORENO Xabier (HOSPITAL BELLVITGE, BARCELONA) ; ALONSO ESCOBAR Maria Nieves (HOSPITAL UNIVERSITARIO DE BADAJOZ, BADAJOZ) ; DEL RÍO GARMA Julio (COMPLEO HOSPITALARIO UNIVERSITARIO DE OURENSE, ORENSE) ; FERNÁNDEZ DOCAMPO Marta (Complejo Hospitalario Universitario de La Coruña, LA CORUÑA) ; VALLES Ana (HOSPITAL SEVERO OCHOA, MADRID) ; CHICA GULLÓN Esther (HOSPITAL DE GETAFE, GETAFE) ; GOMEZ VAZQUEZ Maria Jesus (HOSPITAL DE MOSTOLES, MOSTOLES) ; MARTINEZ NIETO Jorge (HOSPITAL CLINICO SAN CARLOS, MADRID) ; VIEJO LLORENTE Aurora (HOSPITAL UNIVERSITARIO LA PAZ, MADRID) ; MORENO Gemma (HOSPITAL RAMÓN Y CAJAL, MADRID) ; NISTAL GIL Sara (HOSPITAL UNIVERSIARIO MONCLOA, MADRID) ; PAUMARD RODRÍGUEZ Elena (HOSPITAL REINA SOFÍA, MADRID) ; SÁNCHEZ FERNÁNDEZ Mª Soledad (FUNDACIÓN JIMÉNEZ DÍAZ, MADRID) ; GARCÍA CANDEL Faustino (HOSPITAL UNIVERSITARIO VIRGEN DE LA ARRIXACA, MURCIA) ; MARTÍNEZ FRANCÉS Antonio (HOSPITAL UNIVERSITARIO SANTA MARÍA DEL ROSELL, CARTAGENA) ; ANTELO CAAMAÑO Maria Luisa (COMPLEJO HOSPITALARIO DE NAVARRA, PAMPLONA) ; FERNANDEZ ZARZOSO Miguel (HOSPITAL UNIVERSITARIO DOCTOR PESET, VALENCIA) ; GÓMEZ SEGUÍ Inés (HOSPITAL UNIVERSITARIO POLITÉCNICO LA FE, VALENCIA) ; GOTERRIS VICIEDO Rosa (HOSPITAL UNIVERSITARIO CLÍNICO DE VALENCIA, VALENCIA) ; FREIRIA Carmen (HOSPITAL UNIVERSITARIO DE CASTELLON, CASTELLÓN) ; SARMIENTO PALAO Hector (HOSPITAL GENERALUNIVERSITARIO DE ALICANTE, ALICANTE) ; DEL ORBE BARRETO Rafael Andres (HOSPITAL DE CRUCES, BILBAO) ; HERNANDEZ VAZQUEZ Laura (HOSPITAL DE BASURTO, BILBAO) ; PACIELLO CORONEL Maria Liz (HOSPITAL 12 DE OCTUBRE, MADRID) ; LAKWANI Sunil (HOSPITAL UNIVERSITARIO DE TENERIFE – LA LAGUNA, TENERIFE) ; PEREZ Albert (Hospital son espases, MAYORCA) ; DOMINGUEZ ACOSTA Lourdes (Hospital de Jerez, JEREZ DE LA FRONTERA) ; LAVILLA Esperanza (Hospital Lucus Augusti, LUGO) ; RECASENS Valle (Hospital Miguel Servet, ZARAGOZA).

**Members of the Austrian Team:**

Mark Bachner (Department of Medicine, Division Hematology and Oncology, Landesklinikum Wiener Neustadt, Austria), Veronika Buxhofer-Ausch (Department of Internal Medicine I with Hematology, Stem Cell Transplantation, Hemostaseology and Medical Oncology, Ordensklinikum Linz Elisabethinnen, Linz), Kathrin Eller (Division of Nephrology, Department of Internal Medicine, Medical University of Graz, 8036 Graz), Karoline Gleixner (Division of Hematology and Hemostasis, Department of Medicine 1, Medical University of Vienna, Vienna), Paul Knöbl (Division of Hematology and Hemostasis, Department of Medicine 1, Medical University of Vienna, Vienna), Miroslav Krstic (Department of Neurology, Danube-Hospital, Vienna; Wolfgang R. Sperr – Division of Hematology and Hemostasis, Department of Medicine 1, Medical University of Vienna, Vienna, Johannes Thaler (Division of Hematology and Hemostasis, Department of Medicine 1, Medical University of Vienna, Vienna), Martin Ursli (Department of Internal Medicine I, University Hospital St. Pölten, Karl Landsteiner University of Health Sciences).

**Members of the Hungarian team:**

Prohászka Zoltán, Sinkovits György (Research Laboratory, Department of Internal Medicine and Hematology, Semmelweis University, Budapest); Réti Marienn, Várkonyi Andrea, Király Ágnes, Bogsch Luca, Farkas Zita, Reményi Péter (Department of Hematology and Stem Cell Transplantation, Central Hospital of Southern Pest - Institute of Hematology and Infectious Diseases, Budapest); Horváth Laura, Nagy Zsolt, Farkas Péter, Weisinger Júlia (Department of Internal Medicine and Hematology, Semmelweis University, Budapest); Rázsó Katalin (Division of Hematology, Department of Internal Medicine, Faculty of Medicine, University of Debrecen, Debrecen).

**Members of the Canadian team:**

Katerina Pavenski, Megan Buchholz, Amy Moorehead, Michelle Kasimov, St. Michael’s Hospital, Toronto.

**Members of the Japanese team:**

Hisako Kunieda, Department of Hematology, Tokyo Saiseikai Central Hospital, Tokyo; Shinichi Ochi, Atsushi Otani, Shinya Kobayashi, and Hideo Yagi, Department of Hematology and Oncology, Nara Prefecture General Medical Center, Nara, Japan; Kenki Saito, Department of Nara Medical University, Nara; Kana Bando, Department of Hematology, Yokosuka Kyosai Hospital, Kanagawa; Masashi Nishikubo and Hiroki Amagase, Department of Hematology, Kobe City Medical Center General Hospital, Kobe; Ai Matsumoto and Yusuke Yamashita, Department of Hematology/Oncology, Wakayama Medical University, Wakayama.

**Members of the Italian team:**

Pasquale Agosti, Università degli Studi di Milano, Department of Pathophysiology and Transplantation and Fondazione Luigi Villa, Milan, and Fondazione IRCCS Ca' Granda Ospedale Maggiore Policlinico, Angelo Bianchi Bonomi Hemophilia and Thrombosis Center, Milan; Ilaria Mancini, Fondazione IRCCS Ca' Granda Ospedale Maggiore Policlinico, Angelo Bianchi Bonomi Hemophilia and Thrombosis Center, Milan; Flora Peyvandi, Università degli Studi di Milano, Department of Pathophysiology and Transplantation and Fondazione Luigi Villa, Milan, and Fondazione IRCCS Ca' Granda Ospedale Maggiore Policlinico, Angelo Bianchi Bonomi Hemophilia and Thrombosis Center, Milan.

**Member sites of the United States Thrombotic Microangiopathy (USTMA) consortium:**

The Ohio State University, Johns Hopkins University, Baptist Health South Florida, the University of Minnesota, and the University of Kansas.
