# Supplementary material for: Caplacizumab use in immune-mediated thrombotic thrombocytopenic purpura: an international multicentre retrospective Cohort study (The Capla 1000+ project)
Source: eClinicalMedicine. 2025 Mar 30;82:103168. doi: 10.1016/j.eclinm.2025.103168 (PMC11997362; doi:10.1016/j.eclinm.2025.103168)
Supplement: Supplemental Figure S1 [file mmc2.pptx]

## Slide 1
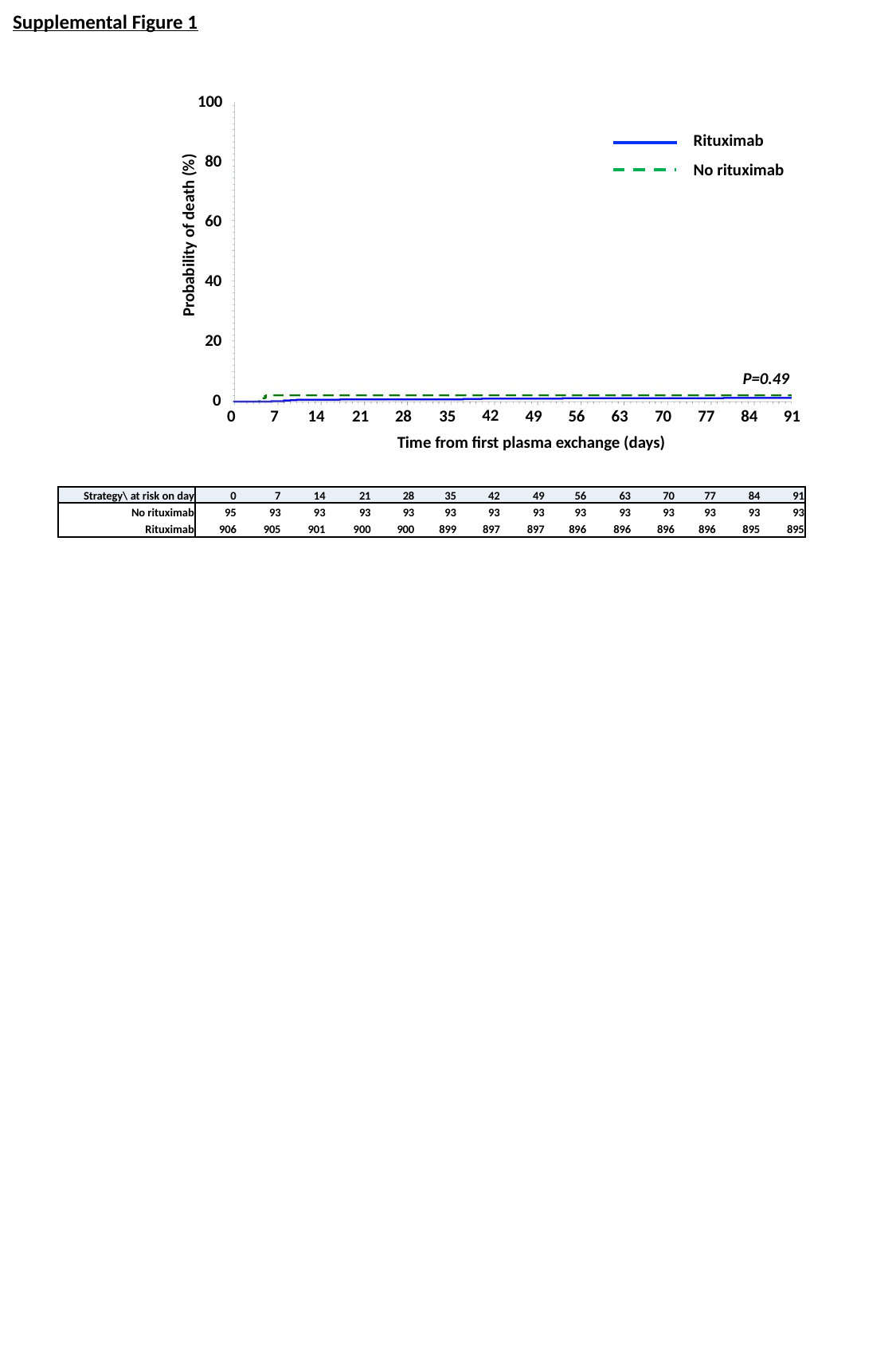

Supplemental Figure 1
100
Rituximab
No rituximab
80
60
Probability of death (%)
40
20
P=0.49
0
42
0
7
14
21
28
35
49
56
63
70
77
84
91
Time from first plasma exchange (days)
| Strategy\ at risk on day | 0 | 7 | 14 | 21 | 28 | 35 | 42 | 49 | 56 | 63 | 70 | 77 | 84 | 91 |
| --- | --- | --- | --- | --- | --- | --- | --- | --- | --- | --- | --- | --- | --- | --- |
| No rituximab | 95 | 93 | 93 | 93 | 93 | 93 | 93 | 93 | 93 | 93 | 93 | 93 | 93 | 93 |
| Rituximab | 906 | 905 | 901 | 900 | 900 | 899 | 897 | 897 | 896 | 896 | 896 | 896 | 895 | 895 |
