# Supplementary material for: Caplacizumab use in immune-mediated thrombotic thrombocytopenic purpura: an international multicentre retrospective Cohort study (The Capla 1000+ project)
Source: eClinicalMedicine. 2025 Mar 30;82:103168. doi: 10.1016/j.eclinm.2025.103168 (PMC11997362; doi:10.1016/j.eclinm.2025.103168)
Supplement: Supplemental Material Capla 1000+280225 [file mmc3.docx]

**1. Supplemental methods**

*Diagnosis and severity assessment*

In a majority of centers (France, Spain, Japan, Germany, Austria, Luxembourg, Brazil, Canada, Hungary, United States and Italy), iTTP diagnosis was considered in patients with features of thrombotic microangiopathy (TMA) and a French score of 1 or 2 or a PLASMIC score of 6-7^1,2^. Patients with a French score of 0 (platelet count ≥30 x 10^3^/mm^3^ and serum creatinine ≥200 µmol/L [2.27 mg/dL]) or a PLASMIC score of <5, were considered as having an alternative diagnosis and were not considered here. The final diagnosis of iTTP was confirmed based on a severe immune-mediated ADAMTS13 deficiency (<10% of activity with positive anti-ADAMTS13 antibodies)^3^. At several centers, iTTP diagnosis was confirmed within 24 hours based on available ADAMTS13 activity assays (UK, Canada [St. Michael’s Hospital], Austria, Hungary, Italy, and some centers in Germany, France and the United States [Kansas, Ohio]). ADAMTS13 activity and anti-ADAMTS13 antibodies were assessed with reference methods as previously reported^4–11^.

iTTP severity was assessed using a severity score based on cerebral involvement (including confusion, stupor, coma or focal deficiency), age, and lactate dehydrogenase (LDH) >10 times the upper normal value level that reflects primarily end organ injury. Patients were then classified into three groups based on their risk of early death^12^. Patients were also classified as having a severe or non-severe disease according to baseline cardiac troponin (*i.e.*, whether it was increased above normal value or not)^13,14^.

*Outcomes*

Assessment of response to treatment was performed according to standard definitions in use during the period of management^15^, with inclusion of some new definitions that became necessary by the use of caplacizumab^16^. Thus, the recent definitions of clinical remission including lacking or only partial ADAMTS13 remission (activity ≥20% but <50%) were considered here^16^ (**Supplemental Table 1**). Patients of the caplacizumab group were compared to those of the control group. Time to clinical response was also specifically assessed in the subgroup of patients with early caplacizumab initiation. The definition of major bleeding and clinically relevant non-major bleeding is taken from the International Society on Thrombosis and Haemostasis (ISTH)^17^.

**Treatment**

TPE was performed daily until platelet count recovery (≥150x10^3^/mm^3^ for at least 24 hours) and stopped with no maintenance TPE, whereas corticosteroids were usually tapered after clinical response, aiming for a total of 3 weeks, as recommended^18^. Patients received usually prednisone orally with a maximal dose of 100 mg/day or methylprednisolone IV 1.0 mg/kg per day. Rituximab (typically 375 mg/m^2^ x4) was administered intravenously on days1-4-8-15 or on a weekly schedule, frontline or as salvage therapy, as per investigator’s choice^18–20^.

**Recruitment of patients of the control group**

Patients of the control cohort were selected consecutively because their management by the participating teams was homogeneous with respect to daily TPE, steroids and rituximab, in accordance with internationally-based recommendations^18^. Data quality control was performed for each control patient; patients with insufficient data quality were replaced by the next consecutive patient until reaching the expected number of controls.

**References**

1 Coppo P, Schwarzinger M, Buffet M, *et al.* Predictive features of severe acquired ADAMTS13 deficiency in idiopathic thrombotic microangiopathies: the French TMA reference center experience. *PloS One* 2010; **5**: e10208.

2 Bendapudi PK, Hurwitz S, Fry A, *et al.* Derivation and external validation of the PLASMIC score for rapid assessment of adults with thrombotic microangiopathies: a cohort study. *Lancet Haematol* 2017; **4**: e157–64.

3 Kremer Hovinga JA, Coppo P, Lämmle B, Moake JL, Miyata T, Vanhoorelbeke K. Thrombotic thrombocytopenic purpura. *Nat Rev Dis Primer* 2017; **3**: 17020.

4 Dutt T, Shaw RJ, Stubbs M, *et al.* Real-world experience with caplacizumab in the management of acute TTP. *Blood* 2021; **137**: 1731–40.

5 Völker LA, Kaufeld J, Miesbach W, *et al.* Real-world data confirm the effectiveness of caplacizumab in acquired thrombotic thrombocytopenic purpura. *Blood Adv* 2020; **4**: 3085–92.

6 Coppo P, Bubenheim M, Azoulay E, *et al.* A regimen with caplacizumab, immunosuppression, and plasma exchange prevents unfavorable outcomes in immune-mediated TTP. *Blood* 2021; **137**: 733–42.

7 Agosti P, De Leo P, Capecchi M, *et al.* Caplacizumab use for immune thrombotic thrombocytopenic purpura: the Milan thrombotic thrombocytopenic purpura registry. *Res Pract Thromb Haemost* 2023; **7**: 102185.

8 de Oliveira Boechat T, de Holanda Farias JS, Ribeiro EFO, de Andrade MLL. Brazilian experience with caplacizumab in acquired thrombotic thrombocytopenic purpura: outcomes of the expanded access program. *Ann Hematol* 2023; **102**: 1581–8.

9 Tse B, Buchholz M, Pavenski K. Management of immune thrombotic thrombocytopenic purpura with caplacizumab: a Canadian, single-centre, real-world experience. *Platelets* 2023; **34**: 2157807.

10 Miyakawa Y, Imada K, Ichikawa S, *et al.* The efficacy and safety of caplacizumab in Japanese patients with immune-mediated thrombotic thrombocytopenic purpura: an open-label phase 2/3 study. *Int J Hematol* 2023; **117**: 366–77.

11 Mingot-Castellano M-E, García-Candel F, Martínez-Nieto J, *et al.* ADAMTS13 recovery in acute thrombotic thrombocytopenic purpura after caplacizumab therapy. *Blood* 2024; **143**: 1807–15.

12 Benhamou Y, Assié C, Boelle P-Y, *et al.* Development and validation of a predictive model for death in acquired severe ADAMTS13 deficiency-associated idiopathic thrombotic thrombocytopenic purpura: the French TMA Reference Center experience. *Haematologica* 2012; **97**: 1181–6.

13 Hughes C, McEwan JR, Longair I, *et al.* Cardiac involvement in acute thrombotic thrombocytopenic purpura: association with troponin T and IgG antibodies to ADAMTS 13. *J Thromb Haemost JTH* 2009; **7**: 529–36.

14 Benhamou Y, Boelle P-Y, Baudin B, *et al.* Cardiac troponin-I on diagnosis predicts early death and refractoriness in acquired thrombotic thrombocytopenic purpura. Experience of the French Thrombotic Microangiopathies Reference Center. *J Thromb Haemost JTH* 2015; **13**: 293–302.

15 Scully M, Cataland S, Coppo P, *et al.* Consensus on the standardization of terminology in thrombotic thrombocytopenic purpura and related thrombotic microangiopathies. *J Thromb Haemost JTH* 2017; **15**: 312–22.

16 Cuker A, Cataland SR, Coppo P, *et al.* Redefining outcomes in immune TTP: an international working group consensus report. *Blood* 2021; **137**: 1855–61.

17 Schulman S, Kearon C. Definition of major bleeding in clinical investigations of antihemostatic medicinal products in non-surgical patients. *J Thromb Haemost JTH* 2005; **3**: 692–4.

18 Zheng XL, Vesely SK, Cataland SR, *et al.* ISTH guidelines for treatment of thrombotic thrombocytopenic purpura. *J Thromb Haemost JTH* 2020; **18**: 2496–502.

19 Scully M, McDonald V, Cavenagh J, *et al.* A phase 2 study of the safety and efficacy of rituximab with plasma exchange in acute acquired thrombotic thrombocytopenic purpura. *Blood* 2011; **118**: 1746–53.

20 Froissart A, Buffet M, Veyradier A, *et al.* Efficacy and safety of first-line rituximab in severe, acquired thrombotic thrombocytopenic purpura with a suboptimal response to plasma exchange. Experience of the French Thrombotic Microangiopathies Reference Center. *Crit Care Med* 2012; **40**: 104–11.

**2. Supplemental File 1.** Details of the dataset defined for the study.

**3. Supplemental File 2**. STROBE methodology checklist.

**4. Supplemental Tables and Figures**

**- Supplemental Table 1.** Definition of outcomes.

**- Supplemental Table 2.** Involved teams, patients’ recruitment and patients previously published.

**- Supplemental Table 3.** Causes of death in iTTP patients of the caplacizumab group.

**- Supplemental Table 4.** Causes of death in iTTP patients of the control group.

**- Supplemental Table 5.** Features at baseline associated with 3-month survival by univariate analysis according to therapeutic groups.

**- Supplemental Table 6.** Details of patients treated with caplacizumab who developed intracranial hemorrhage.

**- Supplemental Figure 1.** Cumulative probability of death after first TPE up to 3 months in patients of the caplacizumab group, stratified by rituximab administration.

**Supplemental Table 1**. Definition of outcomes.

| Clinical response | Full resolution of the neurologic manifestations (or stabilization of neurologic abnormalities in patients considered as having permanent sequels) and renal failure and sustained recovery of normal platelet count (>150x10^9^/L), allowing to stop TPE. |
| --- | --- |
| Clinical remission | Complete response with no further thrombocytopenia or clinical worsening (with no TPE) for more than 30 consecutive days after TPE and caplacizumab was stopped, or if ADAMTS13 activity reaches ≥20%, whichever occurs first. At this step, the episode is considered as ended. |
| Exacerbation | Reappearance of thrombocytopenia, with or without clinical evidence of new ischemic organ injury within 30 days of stopping TPE or caplacizumab, with need to restart treatment. |
| Relapse  ADAMTS13 relapse | Reappearance of thrombocytopenia, with or without clinical evidence of new ischemic organ injury after remission had been achieved (i.e., a new episode of iTTP).  After an ADAMTS13 remission (partial or complete), the ADAMTS13 level decreases again to <20%. |
| Refractoriness | Persistent thrombocytopenia (considered severe if no doubling of baseline platelet count or platelet count < 30x10^9^/L; usually needs salvage therapy) by 4* days of standard treatment, together with persistently elevated LDH levels. |

Abbreviations: TPE: therapeutic plasma exchange; iTTP: immune-mediated thrombotic thrombocytopenic purpura; LDH: lactate dehydrogenase; ADAMTS13: A Disintegrin And Metalloproteinase with ThromboSpondin-1 motifs; member 13; * French and United Kingdom recommendations^6,20^

**Supplemental Table 2.** Involved teams, patients’ recruitment and patients previously published.

|  | **Number of patients** | **Patients previously published** | **References** |
| --- | --- | --- | --- |
| **Caplacizumab group** | **1015** | **396** | |
| France | 400 | 90 | ^1^ |
| United Kingdom | 231 | 81 | ^2^ |
| Germany | 142 | 104 | ^3,4,5,6^ |
| Austria | 14 | 2 | ^3,5^ |
| Luxembourg | 1 | 0 | - |
| Spain | 106 | 77 | ^7^ |
| Italy | 46 | 25 | ^8^ |
| United States | 18 | 1 | ^9^ |
| Japan | 8 | 0 | - |
| Brazil | 6 | 5 | ^10^ |
| Hungary | 12 | 0 | - |
| Canada | 31 | 11 | ^11^ |
|  |  |  |  |
| **Historic controls** | **510** | **421** | |
| France | 215 | 180 | ^1^ |
| United Kingdom | 93 | 39 | ^2^ |
| Germany | 114 | 114 | ^3^ |
| Spain | 48 | 48 | ^12^ |
| United States | 40 | 40 | ^9^ |

**References**

1. Coppo P, Bubenheim M, Azoulay E, Galicier L, Malot S, Bigé N, et al. A regimen with caplacizumab, immunosuppression, and plasma exchange prevents unfavorable outcomes in immune-mediated TTP. Blood. 2021 Feb 11;137(6):733–42.

2. Dutt T, Shaw RJ, Stubbs M, Yong J, Bailiff B, Cranfield T, et al. Real-world experience with caplacizumab in the management of acute TTP. Blood. 2021 Apr 1;137(13):1731–40.

3. Völker LA, Kaufeld J, Balduin G, Merkel L, Kühne L, Eichenauer DA, et al. Impact of first-line use of caplacizumab on treatment outcomes in immune thrombotic thrombocytopenic purpura. J Thromb Haemost JTH. 2023 Mar;21(3):559–72.

4. Völker LA, Kaufeld J, Miesbach W, Brähler S, Reinhardt M, Kühne L, et al. Real-world data confirm the effectiveness of caplacizumab in acquired thrombotic thrombocytopenic purpura. Blood Adv. 2020 Jul 14;4(13):3085–92.

5. Kühne L, Kaufeld J, Völker LA, Wendt R, Schönermarck U, Hägele H, et al. Alternate-day dosing of caplacizumab for immune-mediated thrombotic thrombocytopenic purpura. J Thromb Haemost JTH. 2022 Apr;20(4):951–60.

6. Völker LA, Kaufeld J, Miesbach W, Brähler S, Reinhardt M, Kühne L, et al. ADAMTS13 and VWF activities guide individualized caplacizumab treatment in patients with aTTP. Blood Adv. 2020 Jul 14;4(13):3093–101.

7. Mingot-Castellano ME, García-Candel F, Nieto JM, García-Arroba Peinado J, de la Rubia Comos J, Gomez-Segui I, et al. ADAMTS13 RECOVERY IN ACUTE THROMBOTIC THROMBOCYTOPENIC PURPURA AFTER CAPLACIZUMAB THERAPY. THE SPANISH REGISTRY. Blood. 2024 Jan 18;blood.2023022725.

8. Agosti P, De Leo P, Capecchi M, Ferrari B, Mancini I, Gattillo S, et al. Caplacizumab use for immune thrombotic thrombocytopenic purpura: the Milan thrombotic thrombocytopenic purpura registry. Res Pract Thromb Haemost. 2023 Aug;7(6):102185.

9. Chaturvedi S, Yu J, Brown J, Wei A, Selvakumar S, Gerber GF, et al. Silent cerebral infarction during immune TTP remission: prevalence, predictors, and impact on cognition. Blood. 2023 Jul 27;142(4):325–35.

10. de Oliveira Boechat T, de Holanda Farias JS, Ribeiro EFO, de Andrade MLL. Brazilian experience with caplacizumab in acquired thrombotic thrombocytopenic purpura: outcomes of the expanded access program. Ann Hematol. 2023 Jun;102(6):1581–8.

11. Tse B, Buchholz M, Pavenski K. Management of immune thrombotic thrombocytopenic purpura with caplacizumab: a Canadian, single-centre, real-world experience. Platelets. 2023 Dec;34(1):2157807.

12. Izquierdo CP, Mingot-Castellano ME, Fuentes AEK, García-Arroba Peinado J, Cid J, Jimenez MM, et al. Real-world effectiveness of caplacizumab vs the standard of care in immune thrombotic thrombocytopenic purpura. Blood Adv. 2022 Dec 27;6(24):6219–27.

**Supplemental Table 3.** Reported causes of death in iTTP patients of the caplacizumab group (N=15).

| **Cause of death** | **Age (yo)** | **Context of death** |
| --- | --- | --- |
| Deaths directly related to uncontrolled iTTP (N=8) | 71  65  62  54  61  58  43  76 | Exacerbation with massive cardiac involvement and deterioration related to TTP (had baseline significant coronary artery disease).  Worsened multi-organ failure before initial management.  Intestinal gangrene with sepsis.  Cardiac arrest and cardiopulmonary resuscitation due to ST elevation myocardial infarction and extensive cerebral infarction with bleeding.  Multiorgan failure.  No electro-encephalographic activity.  Pulseless electrical activity; cardiac arrest.  Cardiac arrest. |
| Deaths resulting from iTTP-related comorbidities (N=4)* | 83  66  79  67 | Severe bilateral pulmonary emboli with cardiac arrest.  Discharged to palliative care post-massive ischemic stroke of MCA while iTTP was in clinical response.  Death 7 weeks following transfer to local hospital, due to multiple comorbidities.  Intracerebral hemorrhage post-ischemic infarction while in clinical response. |
| Death considered not directly related to iTTP (N=3) | 74  68  56 | Fatal SARS-COV-2 infection.  Fatal anaphylaxis to plasma.  Anoxic encephalopathy due to cardiac arrest post-traumatic sternal bone marrow aspirate. |

Abbreviations: iTTP: immune-mediated thrombotic thrombocytopenic purpura; yo: year-old; MCA: middle cerebral artery. SARS-COV-2: Severe Acute Respiratory Syndrome-Coronavirus-2. *iTTP was in clinical response.

**Supplemental Table 4.** Reported causes of death in iTTP patients of the historic control group (N=30).

| **Age (yrs)** | **Context of death** |
| --- | --- |
| 43 | Refractory iTTP; death during salvage splenectomy. |
| 65 | Multiorgan failure; hemorrhagic and septic shock post-surgery. |
| 38 | Multiorgan failure. |
| 73 | Arrhythmia and cardiorespiratory arrest |
| 62 | Sudden neurologic worsening. |
| 44 | Neurologic worsening during 7th TPE and cardiorespiratory arrest. |
| 49 | Multiorgan failure. |
| 55 | Multiorgan failure. |
| 46 | Multiorgan failure and cardiorespiratory arrest. |
| 84 | Multiorgan failure. |
| 60 | Multiorgan failure. |
| 63 | Multiorgan failure and cardiorespiratory arrest. |
| 49 | Multiorgan failure and cardiorespiratory arrest. |
| 65 | Multiorgan failure. |
| 32 | Hemorrhagic shock. |
| 39 | Sudden neurologic deterioration at first TPE; then cardio-respiratory arrest. |
| 77 | Sudden neurologic deterioration. |
| 54 | Multiorgan failure. |
| 60 | Multiorgan failure. |
| 50 | Multiorgan failure. |
| 33 | Multiorgan failure. |
| 36 | Multiorgan failure. |
| 64 | NA. |
| 52 | NA. |
| 27 | Myocardial infarction, then multiorgan failure. |
| 81 | NA. |
| 31 | NA. |
| 52 | Multiorgan failure. |
| 57 | Pulseless electrical activity, cardiac arrest. |
| 83 | Multiorgan failure. |

Abbreviations: iTTP: immune-mediated thrombotic thrombocytopenic purpura; yo: year-old; TPE: therapeutic plasma exchange; NA: not available.

**Supplemental Table 5.** Features at baseline associated with 3-month survival by univariate analysis according to therapeutic groups.

|  | **Caplacizumab group** | | | **Control group** | | |
| --- | --- | --- | --- | --- | --- | --- |
|  | **Survivors**  **(N=1000)** | **Non-survivors**  **(N=15)** | **OR (95%CI OR)**  **p-value** | **Survivors**  **(N=480)** | **Non-survivors**  **(N=30)** | **OR (95%CI OR)**  **p-value** |
| **French severity score features** |  |  |  |  |  |  |
| Age (yo)    ≤40 yo  40-60 yo  >60 yo | 45 (33-57)  402 (40%)  400 (40%)  198 (20%) | 66 (59.5-72.5)  0  4 (27%)  11 (73%) | 1.08 (1.04-1.12)^£^  <0.0001 | 43 (33-56)  201 (42%)  202 (42%)  77 (16%) | 53 (43-64)  7 (23%)  13 (44%)  10 (33%) | 1.03 (1.01-1.06)^£^  0.002 |
| Neurologic involvement | (N=966)  483 (50%) | (N=15)  10 (67%) | 2.00 (0.71-6.47)  0.20 | (N=405)  166 (41%) | (N=28)  16 (57%) | 1.94 (0.90-4.30)  0.09 |
| LDH >10x upper normal value | (N=916)  44 (4.8%) | (N=15)  0 | 0 (0-2.45)  0.20 | (N=480)  26 (5.4%) | (N=30)  4 (13.3%) | 3.30 (0.91-9.56)  0.07 |
| French severity score*  Low (0-1)  Intermediate (2)  High (3-4) | (N=874)  502 (58%)  243 (27%)  129 (15%) | (N=15)  2 (13%)  5 (33%)  8 (54%) | 0.06 (0.01-0.31)  0.33 (0.11-1.04)  6.5 (2.0-22)  <0.001 | (N=354)  226 (64%)  90 (25%)  38 (11%) | (N=27)  11 (41%)  7 (26%)  9 (33%) | 0.21 (0.08-0.61)  0.30 (0.09-1.01)  4.1 (1.52-10.5)  0.02 |
| **Troponin** |  |  |  |  |  |  |
| Troponin >upper normal value | (N=768)  545 (71%) | (N=13)  12 (92%) | 4.80 (0.62-37)  0.13 | (N=307)  205 (67%) | (N=22)  20 (91%) | 5.0 (1.14-22)  0.03 |

Abbreviations: OR (95%CI): Odds-Ratio with 95% confidence interval for Odds-Ratio; yo: year-old; LDH: Lactate dehydrogenase. *Patients at high risk of early death of iTTP were defined by a severity score ≥3 (cerebral involvement: yes=1 / no=0, LDH: >10xULN=1 / ≤10xULN=0, age: >60 years=2 / >40 and ≤60 years=1 / ≤40 years=0)^7^. ^£^OR per unit. Qualitative variables are provided as percentage of patients with available data in the respective treatment group with valid data. P-value was considered significant when <0.05.

**Supplemental Table 6.** Features at baseline associated with 3-month survival by univariate analysis.

|  | **Survivors**  **(N=1480)** | **Non-survivors**  **(N=45)** | **OR (95%CI OR)**  **p-value** |
| --- | --- | --- | --- |
| **French severity score features** |  |  |  |
| Age (yo)    ≤40 yo  40-60 yo  >60 yo | 44 (33-57)  603 (41%)  602 (41%)  275 (18%) | 60 (49-67)  7 (15%)  17 (38%)  21 (47%) | 1.05 (1.03-1.07)^£^  <0.0001 |
| Neurologic involvement | (N=1375)  649 (47%) | (N=43)  26 (60%) | 1.71 (0.92-3.18)  0.09 |
| LDH >10x upper normal value | (N=925)  83 (9%) | (N=4)  38 (10.5) | 1.19 (0.3-3.47)  0.77 |
| French severity score*  Low (0-1)  Intermediate (2)  High (3-4) | (N=1228)  728 (59%)  333 (27%)  167 (14%) | (N=36)  11 (30%)  10 (28%)  15 (42%) | 0.17 (0.076-0.37)  0.33 (0.15-0.76)  4.53 (2.12-9.4)  <0.0001 |
| **Troponin** |  |  |  |
| Troponin >upper normal value | (N=1070)  750 (70%) | (N=35)  32 (91%) | 4.55 (1.38-15)  0.01 |
| **Treatment** |  |  |  |
| Caplacizumab | (N=1480)  993 (67.4%) | (N=45)  15 (33%) | 4.14 (2.2-7.76)  <0.0001 |
| Corticosteroids | (N=1480)  1447 (98%) | (N=45)  39 (87%) | 6.7 (2.65-16.9)  <0.0001 |
| Rituximab | (N=1468)  1241 (85%) | (N=45)  15 (64%) | 3.01 (1.6-5.6)  <0.001 |

Abbreviations: OR (95%CI): Odds-Ratio with 95% confidence interval for Odds-Ratio; yo: year-old; LDH: Lactate dehydrogenase. *Patients at high risk of early death of iTTP were defined by a severity score ≥3 (cerebral involvement: yes=1 / no=0, LDH: >10xULN=1 / ≤10xULN=0, age: >60 years=2 / >40 and ≤60 years=1 / ≤40 years=0)^7^. ^£^OR per unit. Qualitative variables are provided as percentage of patients with available data in the respective groups with valid data. P-value was considered significant when <0.05.

**Supplemental Table 7.** Features at baseline associated with 3-month survival by multivariate analysis.

|  | **OR (95%CI OR)**  **p-value** |
| --- | --- |
| Age (yo) | 1.06 (1.03-1.09)^£^  <0.0001 |
| Caplacizumab | 3.00 (1.37-6.98)  <0.01 |

Abbreviations: OR (95%CI): Odds-Ratio with 95% confidence interval for Odds-Ratio; yo: year-old; ^£^OR per unit. P-value was considered significant when <0.05.

**Supplemental Table 8.** Details of patients treated with caplacizumab who developed intracranial hemorrhage.

| **Patient #** | **Age (yrs)** | **Context** |
| --- | --- | --- |
| 1 | 67 | Male - Developed a fatal ICH late in the management, while iTTP was in clinical response |
| 2 | 64 | Female - ICH was found on cerebral magnetic resonance imaging two days after caplacizumab initiation. The patient recovered uneventfully after caplacizumab interruption with continued TPE and immunosuppression. |
| 3 | 51 | Female - Developed a spontaneous subdural hematoma leading to withdrawal of caplacizumab. The patient subsequently experienced an exacerbation while ADAMTS13 was still undetectable, which was treated successfully with recombinant ADAMTS13. |

Abbreviations: ICH: intracranial hemorrhage; iTTP: immune-mediated thrombotic thrombocytopenic purpura; TPE: therapeutic plasma exchange; ADAMTS13: A Disintegrin And Metalloproteinase with ThromboSpondin-1 motifs; member 13.
